# Horizontal gene transfer and nucleotide compositional anomaly in large DNA viruses

**DOI:** 10.1186/1471-2164-8-456

**Published:** 2007-12-10

**Authors:** Adam Monier, Jean-Michel Claverie, Hiroyuki Ogata

**Affiliations:** 1 Structural and Genomic Information Laboratory, CNRS – UPR 2589, Institute for Structural Biology and Microbiology, Parc Scientifique de Luminy, 163 avenue de Luminy, FR-13288, Marseille cedex 09, France

## Abstract

**Background:**

DNA viruses have a wide range of genome sizes (5 kb up to 1.2 Mb, compared to 0.16 Mb to 1.5 Mb for obligate parasitic bacteria) that do not correlate with their virulence or the taxonomic distribution of their hosts. The reasons for such large variation are unclear. According to the traditional view of viruses as gifted "gene pickpockets", large viral genome sizes could originate from numerous gene acquisitions from their hosts. We investigated this hypothesis by studying 67 large DNA viruses with genome sizes larger than 150 kb, including the recently characterized giant mimivirus. Given that horizontally transferred DNA often have anomalous nucleotide compositions differing from the rest of the genome, we conducted a detailed analysis of the inter- and intra-genome compositional properties of these viruses. We then interpreted their compositional heterogeneity in terms of possible causes, including strand asymmetry, gene function/expression, and horizontal transfer.

**Results:**

We first show that the global nucleotide composition and nucleotide word usage of viral genomes are species-specific and distinct from those of their hosts. Next, we identified compositionally anomalous (cA) genes in viral genomes, using a method based on Bayesian inference. The proportion of cA genes is highly variable across viruses and does not exhibit a significant correlation with genome size. The vast majority of the cA genes were of unknown function, lacking homologs in the databases. For genes with known homologs, we found a substantial enrichment of cA genes in specific functional classes for some of the viruses. No significant association was found between cA genes and compositional strand asymmetry. A possible exogenous origin for a small fraction of the cA genes could be confirmed by phylogenetic reconstruction.

**Conclusion:**

At odds with the traditional dogma, our results argue against frequent genetic transfers to large DNA viruses from their modern hosts. The large genome sizes of these viruses are not simply explained by an increased propensity to acquire foreign genes. This study also confirms that the anomalous nucleotide compositions of the cA genes is sometimes linked to particular biological functions or expression patterns, possibly leading to an overestimation of recent horizontal gene transfers.

## Background

During the last decade the study of virus evolution has been neglected to the point where 'virus evolution' most often refers to studies more akin to population genetics, such as the worldwide scrutiny of new polymorphisms appearing daily in the H5N1 avian flu virus [1], than to the fundamental question of where viruses come from [2-4]. Phylogenetic studies on viruses have long been considered unfeasible for two main reasons: 1) their reputed propensity to randomly acquire genetic material from their host or the environment and 2) their reputed very high sequence divergence rate. The generality of this vision, probably inherited from the study of RNA viruses (in particular retroviruses), now deserves to be revisited for DNA viruses in light of the increasing amount of available genomic sequence data, and the recent characterization of some giant viruses [5-8].

When analyzing DNA virus genome sequences on a global scale [9] one is immediately struck by their tremendous variation in size. Even if viral DNA genomes are expected to be larger than viral RNA genomes due to the improved accuracy of the replication system, it is not as easy to understand how DNA viruses with apparently similar "fitness" (as judged from their virulence and burst sizes) may have come to exhibit sizes ranging from a few kilobases up to more than a megabase [5,6].

Even more intriguing is the fact that such a genome size variation is commonly found among viruses infecting the same or similar hosts, for prokaryotic viruses (e.g. bacteriophage ranging from 30 kb up to nearly 670 kb for bacteriophage G [10]), as well as animal viruses [from less than 5 kb (polyomaviruses) to 360 kb (poxviruses)]. Currently the largest known eukaryotic DNA viruses are plankton parasite *Emiliania huxleyi *virus 86 (407 kb) [7] and the amoeba-infecting mimivirus (1.2 Mb) [5].

Finally, unlike the situation for eukaryotic cellular organisms, the increase in viral genome size is not correlated with either accumulation of "junk" DNA (e.g. low complexity sequences or non-coding regions), invasion of mobile elements, gene duplication or repeat expansion [5,7,11].

In the present work, we now examine to which extent horizontal gene transfer (HGT) from host might account for the exceptional genome size of several families of large double stranded DNA viruses (LDVs) with genomes exceeding 150 kb in size.

These viruses are found in a wide variety of viral families including those classified in Nucleo-Cytoplasmic Large DNA viruses [12] (*Asfarviridae*, *Poxviridae*, *Phycodnaviridae*, *Iridoviridae*, *Mimiviridae*) as well as herpesviruses, nimaviruses, baculoviruses, and bacteriophages. At the time of this study, 67 LDV genomes (>150 kb) were available in public databases (Table 1). Each of these genomes encodes hundreds of predicted protein-coding genes. With this increasing body of data, it has now become possible to analyze different structural and functional aspects of those LDV genomes. Note that polydnaviruses were not analyzed due to their anomalous low gene density, atypical chromosomal organization and life style [13].

**Table 1:** Proportions of cA genes in the 67 large DNA viruses.

**Classification**	**Virus**	**Genome size (bp)**	**Genomic G+C (%)**	**Number of analyzed CDS**	**Number of cA genes**	**Proportions of cA genes (%)**	**Co-localization**^a^
Caudovirales	Enterobacteria phage RB43	180500	43.2	177	15	8.47	-
	Enterobacteria phage T4	168903	35.3	175	8	4.57	-
	*Pseudomonas *phage phiEL	211215	49.33	178	18	10.11	-
	*Pseudomonas *phage phiKZ	280334	36.83	278	27	9.71	-
	*Mycobacterium *phage Bxz1	156102	64.77	147	27	18.37	-
	Enterobacteria phage RB69	167560	37.66	160	11	6.88	*
	Enterobacteria phage RB49	164018	40.44	160	12	7.5	-
	*Vibrio *phage KVP40	244835	42.6	256	18	7.03	-
	*Aeromonas *phage 44RR2.8t	173591	43.88	173	7	4.05	-
	*Aeromonas *phage Aeh1	233234	42.78	228	13	5.7	-
	Bacteriophage S-PM2	196280	37.82	135	28	20.74	*
	Cyanophage P-SSM2	252401	35.51	212	33	15.57	*
	Cyanophage P-SSM4	178249	36.74	121	20	16.53	**
	*Aeromonas *phage 31	172963	43.91	173	10	5.78	-
	Bacteriophage c-st	185683	26.28	151	8	5.3	-
Alphaherpesvirinae	Cercopithecine herpesvirus 16	156487	76.09	71	18	25.35	-
	Equid herpesvirus 1	150224	56.67	79	12	15.19	-
	Cercopithecine herpesvirus 1	156789	74.46	71	23	32.39	-
	Human herpesvirus 2	154746	70.39	72	21	29.17	*
	Human herpesvirus 1	152261	68.28	72	24	33.33	-
	Psittacid herpesvirus 1	163025	60.95	71	8	11.27	-
	Gallid herpesvirus 2	174077	43.89	106	19	17.92	*
	Gallid herpesvirus 3	164270	53.61	92	19	20.65	***
	Cercopithecine herpesvirus 2	150715	75.97	71	22	30.99	*
	Meleagrid herpesvirus 1	159160	47.56	85	8	9.41	***
Betaherpesvirinae	Pongine herpesvirus 4	241087	61.7	155	67	43.23	***
	Human herpesvirus 6B	162114	42.77	91	6	6.59	-
	Murid herpesvirus 1	230278	58.73	156	52	33.33	***
	Human herpesvirus 5 strain AD169	230287	57.19	145	53	36.55	***
	Human herpesvirus 6	159321	42.44	107	11	10.28	-
	Human herpesvirus 7	153080	36.22	78	8	10.26	-
	Cercopithecine herpesvirus 8	221454	49.14	223	80	35.87	-
	Murid herpesvirus 2	230138	61.01	165	87	52.73	***
	Human herpesvirus 5 strain Merlin	235645	57.48	153	56	36.6	***
	Tupaiid herpesvirus 1	195859	66.61	144	59	40.97	***
Gammaherpesvirinae	Human herpesvirus 4	171823	59.5	90	37	41.11	*
	Equid herpesvirus 2	184427	57.5	74	28	37.84	-
	Cercopithecine herpesvirus 15	171096	61.94	77	22	28.57	*
Herpesvirinae Uncl.	Ostreid herpesvirus 1	207439	38.73	121	7	5.79	-
Chordopoxvirinae	Monkeypox virus	196858	33.09	162	3	1.85	-
	Camelpox virus	205719	33.17	171	6	3.51	**
	Cowpox virus	224499	33.4	189	2	1.06	-
	Myxoma virus	161773	43.56	147	5	3.4	-
	Rabbit fibroma virus	159857	39.53	138	4	2.9	-
	Ectromelia virus	209771	33.18	154	6	3.9	-
	Variola virus	185578	32.73	152	6	3.95	-
	Molluscum contagiosum virus	190289	63.36	139	43	30.94	***
	Canarypox virus	359853	30.37	291	14	4.81	-
	Fowlpox virus	288539	30.89	230	12	5.22	-
	Lumpy skin disease virus	150773	25.91	136	4	2.94	-
	Vaccinia virus	194711	33.34	179	3	1.68	-
Entomopoxvirinae	*M. sanguinipes *entomopoxvirus	236120	18.27	208	15	7.21	-
	*A. moorei *entomopoxvirus 'L'	232392	17.78	218	16	7.34	-
Poxvirinae Uncl.	Mule deer poxvirus	166259	26.16	148	2	1.35	-
Asfarviridae	African swine fever virus	170101	38.95	129	22	17.05	**
Baculovirviridae	*M. configurata *NPV-A	155060	41.68	146	7	4.79	-
	*M. configurata *NPV-B	158482	40.04	146	7	4.79	-
	*L. dispar *MNPV	161046	57.47	133	34	25.56	-
	*Xestia c-nigrum *granulovirus	178733	40.68	145	8	5.52	-
Iridoviridae	Lymphocystis disease virus	186250	27.23	127	7	5.51	-
	Invertebrate iridescent virus 6	212482	28.63	178	15	8.43	-
Mimiviridae	*A. polyphaga *mimivirus	1181404	27.96	910	83	9.12	-
Phycodnaviridae	*E. huxleyi *virus 86	407339	40.18	416	44	10.58	-
	*P. bursaria *Chlorella virus 1	330743	39.97	426	117	27.46	-
	*E. siliculosus *virus 1	335593	51.73	209	25	11.96	-
Nimaviridae	Shrimp white spot syndrome virus	305107	41.01	245	96	39.18	-
Unclassified Virus	*H. zea *virus 1	228089	41.86	104	25	24.04	*

*a*. '-' no significance; * *p *≤ 0.05; ** *p *≤ 0.01; *** *p *≤ 0.001.

Nucleotide composition is one of the specific properties of viral and cellular genomes [14-16]. The mimivirus genome is A+T rich (72%), and exhibits rather homogenous nucleotide compositions along the chromosome [5]. In contrast, some betaherpesviruses exhibit a clear bimodal heterogeneity in G+C composition along their genomes [17]. Different factors can shape compositional heterogeneity within and across genomes [18], including mutational biases, physical constraints on DNA molecules, functional requirements at the level of transcription [19] and translation [20-22], and genetic exchanges with other genomes by horizontal transfer [23-26]. Many of the initial surveys of LDV genomes characterized nucleotide compositional properties of individual genomes. However, there are few studies systematically addressing their compositional properties in a comparative way [27].

Here we analyzed the nucleotide compositional properties of 67 LDV genomes. We first compared global nucleotide compositions across these viruses and their hosts. We next identified compositionally anomalous (cA) genes in the viral genomes, examined their correlation with strand asymmetry, a possible cause of compositional biases, and described their functional and physical (i.e. chromosomal co-localization) properties. Finally, we investigated potential exogenous origins of the cA genes through phylogenetic tree reconstruction.

## Results

### Global compositional bias differs across LDVs and their hosts

G+C content is a simple measure of genomic nucleotide composition, and it has been shown to be species-specific for prokaryotes [15]. LDVs also present a large variation in global G+C content across viral families (27%–76%; Additional file 1). Large variations in G+C content are also observed within a viral family or subfamily (Additional file 2), for instance, the lumpy skin disease virus (26%) and the molluscum contagiosum virus (64%) belonging to the same chordopoxvirus subfamily. Large variations in G+C content were previously noted for herpesviruses [28].

**Figure 1:**
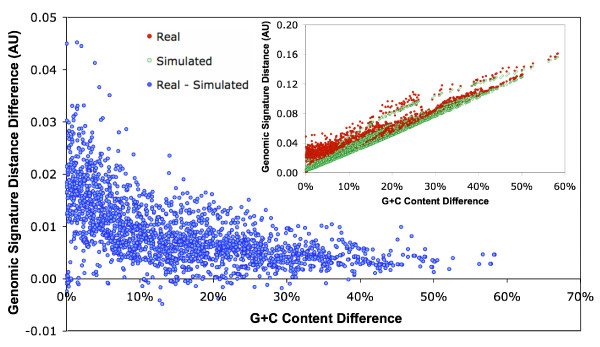
**Difference in genomic signature distance between real and randomized data for all possible pairs of 67 LDVs**. The inset figure shows the genomic signature distances between all possible pairs of 67 LDVs for both real and randomized data.

Nucleotide word frequency is a finer indicator of genomic specificity [29]. We computed the frequencies of tetra-nucleotide words in the LDV genomes (i.e. genomic signatures [30]) and genomic signature distances for all possible pairs of the 67 LDVs (see **Methods**). The genomic signature distance was strongly correlated with the difference in G+C content (R^2 ^= 0.92) as a consequence of the important role of G+C content in shaping nucleotide word frequencies (Fig. 1). However, we observed a large variation in the genomic signature distance for similar differences in G+C contents. This variation suggests the existence of higher order compositional biases that are independent of G+C content. We shuffled every LDV genome sequence to erase tetra-nucleotide word biases that were independent of base compositions, and re-computed the genomic signature distances for all the pairs of the 67 randomized sequences. We observed a significant deviation in the genomic signature distances between the real and the randomized data (*t*-test, *p *< 0.001; Fig. 1). Thus LDVs maintain species-specific global compositional biases at both single and tetra-nucleotide levels, as previously observed for cellular organisms [30] and for a few viral families [28]. In other words, these LDVs have a certain level of homogeneity in nucleotide composition within their genomes, as Mrázek and Karlin previously suggested for eighteen large DNA viruses [27].

**Figure 2:**
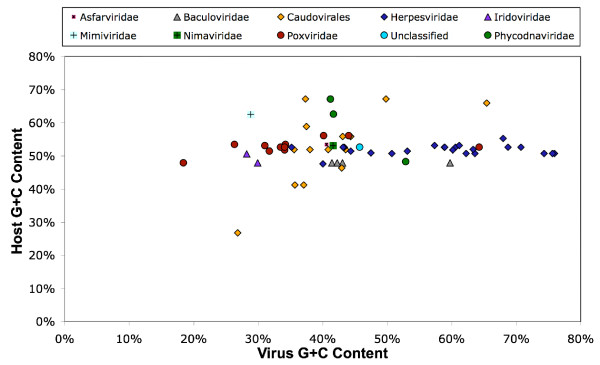
Comparison of G+C content between LDVs and their hosts.

**Figure 3:**
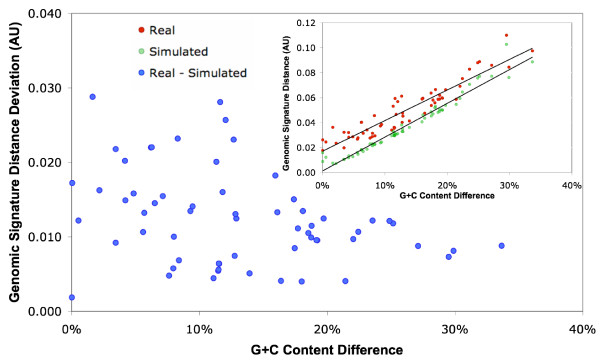
**Difference in genomic signature distance between real and randomized data for LDV-host pairs**. The inset figure shows the genomic signature distances between the LDV-host pairs for both real and randomized data.

Viruses replicate intracellularly in their hosts, using the host nucleotide pool. One may expect that the global nucleotide compositional biases in viral genomes are similar to those of their hosts [31,32]. Host genomes are also potential sources of genes for viruses, as viral genomes are known to incorporate host genes. Thus the comparison of the nucleotide compositional properties between LDVs and their hosts is of particular interest. We compared nucleotide compositional biases between the LDVs and their hosts (i.e. modern hosts or their close relatives). We observed a clear lack of correlation between the G+C content of the coding regions, (G+C)_CDS_, of the LDV genomes, and the (G+C)_CDS _of the cellular host genomic sequences (Fig. 2). A genomic signature analysis also revealed distinct tetra-nucleotide word preferences between LDVs and their hosts independent of their base composition differences (Fig. 3). After a base-by-base random shuffling of the analyzed sequences, we observed a significant deviation between the real and the randomized data in the genomic signature distances for the LDV-host pairs (*t*-test, *p *< 0.001; Fig. 3). Thus the species-specific global compositional biases of LDVs are distinct from that of their hosts. This invalidates the concept that viruses tend to adapt their global DNA signatures to the host machinery. Furthermore, HGTs from their modern hosts are not as frequent, if any, as they could have influences on global nucleotide compositions of LDV genomes.

#### Compositionally anomalous genes in LDVs

We next analyzed compositional heterogeneities across different genes encoded in individual LDV genomes. Genes with nucleotide compositions substantially deviating from the average in a genome tend to be under distinct selection pressures or have particular evolutionary histories. We denote such genes as compositionally anomalous (cA) genes. To identify cA genes with a robust statistical support, we used a method [26] based on Markov modeling and Bayesian inference originally developed for the identification of horizontally transferred genes within prokaryotic genomes. Composition-based approaches have a clear limitation in detecting HGTs; they detect only a subset of HGTs (i.e. recent horizontal gene acquisitions by a recipient genome from a donor genome with nucleotide compositions that are different from the recipient genome). Our aim here is to examine the nature of cA genes in LDVs in the light of previous observations of cA genes for prokaryotic genomes.

**Figure 4:**
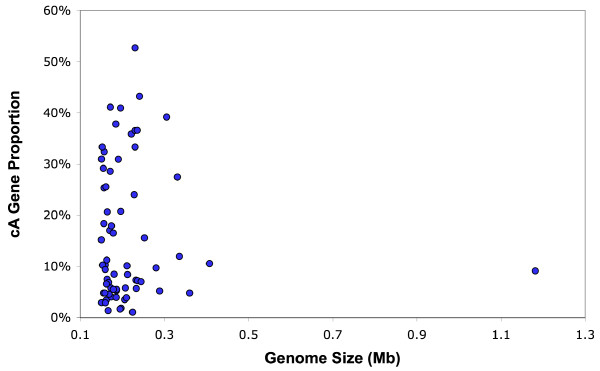
Proportions of the cA genes detected in the 67 LDV genomes, compared with the genome sizes.

We identified cA genes in all the analyzed LDV genomes. In many LDV genomes, the cA genes were more A+T rich than the remaining genes (for 43 out of 67 LDVs; binomial test, *p *< 0.05). The proportion of cA genes per genome was highly variable across the 67 LDVs, ranging from 1% to 53% (Table 1, Fig. 4). In contrast, the proportion obtained by the same method is less variable for prokaryotes (0% to 25%) [9]. Herpesviruses exhibited the widest range of cA gene proportion (6% to 53%). All but two of the analyzed NCLDVs, including mimivirus, had a relatively low level of cA proportion (1%–17%). Two exceptions were *Paramecium bursaria *chlorella virus 1 (28%) and molluscum contagiosum virus (31%). Phages showed a relatively low proportion (4% to 21%). The cA gene proportions for other viruses were 5% to 26% for four baculoviruses, 39% for nimavirus and 24% for *Heliothis zea *virus 1. The very high cA proportions in some betaherpesviruses appear to be linked with genomic islands harboring betaherpesvirus-specific genes, which we will discuss below (in the "Co-localization of cA genes" section).

Nakamura *et al. *reported a significant positive correlation between the cA gene proportion and the total number of genes in a genome for prokaryotes [26]; larger prokaryotic genomes tend to more rapidly acquire foreign genes (of "anomalous nucleotide compositions") than smaller ones. In contrast, we found no such correlation for the 67 LDVs (R^2 ^= 0.03) (Additional file 3).

We observed a weak but significant positive correlation (R^2 ^= 0.53, *p *< 0.005) between the proportion of cA genes and genomic G+C content (Additional file 4). This correlation was not due to a bias induced by a given viral family or subfamily. For example, when we excluded herpesviruses, many of which exhibit both a high cA gene proportion and a high G+C content, the correlation was reduced but still remained significant (R^2 ^= 0.24, *p *≤ 0.005). Such a significant correlation was observed also within a single subfamily (i.e. alphaherpesviruses, R^2 ^= 0.66, *p *≤ 0.005). A potential variation in gene prediction quality (which could be dependent on genomic G+C compositions) does not appear to explain these correlations (Monier *et al.*, unpublished data). We also examined a possible relationship between the cA gene proportion and chromosome topology. There was no difference in the cA gene proportions between linear LDV genomes (15.5% on average) and circular LDV genomes (17.3% on average; *t*-test, *p *= 0.63).

Our global genomic signature analysis revealed remarkably different nucleotide compositions between LDVs and their hosts. To investigate further the discrepancy of nucleotide compositions, we examined if the identified cA genes are enriched in "host-like" sequences in terms of their nucleotide compositions. We determined the genomic signature distances (di-nucleotides for word length) from individual cA genes to the viral genome where they originate as well as to the host genomic sequences. As shown in the Additional file 5, we observed a marked enrichment of genes with host-like signatures among the identified cA genes (19%) relative to non-cA genes (3.3%). The host-like signatures in these cA genes could be due to a large variation in their nucleotide compositions, being unrelated to possible host origins for some of the cA genes. To test this hypothesis, we performed the same analysis by randomizing the pairings between viruses and their hosts. A comparable fraction (23% versus 19%) of the cA genes indeed exhibited smaller distances to the genomic signatures of randomly chosen hosts than to the viral genomes where they originate. Thus the enrichment of genes with host-like signatures in cA genes may not suggest their horizontal acquisitions from hosts.

#### Correlation between cA genes and replication-associated strand asymmetry

Genome sequences of many prokaryotes and vertebrates [33,34] show strong strand asymmetry in nucleotide composition between the leading and the lagging strands. For most bacteria, compositional strand asymmetry is characterized by an excess of G and T bases in the leading strands relative to the lagging strands [33,35,36]. A substantial part of compositional strand asymmetry is independent of gene distribution in the two distinct DNA strands, and is probably due to mutational biases linked to asymmetric mechanisms of DNA replication [37,38]. Compositional strand asymmetry spanning large genomic segments has been described also for some large DNA viruses, including mimivirus [5,39] and several herpesviruses [40,41]. Such replication-associated strand bias can potentially result in two classes of genes with distinct nucleotide compositions, depending on which strand they are located. We thus examined if there is a correlation between the distribution of cA genes and compositional strand asymmetry.

We first generated cumulative GT-excess plots [42] for all of the 67 LDV genomes to assess their compositional strand asymmetry. LDVs presented various GT-excess patterns. Several LDVs, mostly phages, showed strong global strand asymmetry with a monotonous increase (or decrease) of the cumulative GT-excess curve along their entire genome. Some LDVs including mimivirus exhibited a few peaks in their GT-excess profiles. For other LDVs, the GT-excess curves locally fluctuated with no long genomic segments exhibiting a consistent compositional asymmetry. We selected fifteen LDVs presenting long (>10 kb) genomic segments with uniform compositional asymmetry (Additional file 6). After identifying the genomic coordinates where the sign of the nucleotide-skew (G+T *versus *C+A) changes, we split the genomic sequences into sub-strands. Those sub-strands were classified into two classes: class I consists of sub-strands having a positive nucleotide-skew (i.e. G+T% > C+A%), and class II with a negative skew (i.e. G+T% < C+A%). Genes were then classified into either class I or II according to the sub-strand they originated from. For instance, the plus strand (according to the GenBank entry) of the *Pseudomonas *phage phiKZ genome is G and T rich along its entire length, thus being classified as class I. This class I strand contains 229 genes (≥ 300 nt), of which 22 were detected as cA genes. The complementary strand is classified as a class II and contains 49 genes, of which 5 were cA genes. No significant correlation was found between the distribution of cA genes and the compositional strand asymmetry (Fisher's exact test, *p*-value = 0.99). The mimivirus genome shows a clear switch of nucleotide-skew (G+T *versus *C+A) at position 380,000 nt as previously noted [5]. Thus a part of the plus strand (from 0 to 380,000 nt) and a part of the complementary strand (from 380,000 to 1,118,404 nt) constitute the class I strands. Class II is represented by the remaining strands (Additional file 6). Again, no significant correlation was found between the distribution of cA genes and the compositional strand asymmetry (*p*-value = 0.63). Of the fifteen LDVs that we analyzed, only three showed a significant correlation between cA gene distribution and strand asymmetries (*p*-values < 0.05). The three viruses are the fowlpox virus and two strains of the human herpesvirus 5 (the wild type strain merlin and the laboratory strain AD169). These results suggest that replication-associated compositional strand asymmetry accounts for only a small part of the nucleotide compositional biases of the cA genes observed in LDVs.

#### Co-localization of cA genes

**Figure 5:**
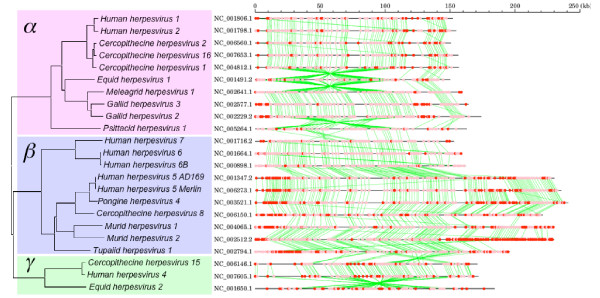
**Gene organization and the locations of the cA genes for herpesviruses**. The phylogenetic reconstruction (on the left) is based on the DNA polymerase catalytic subunit sequences. Red and pink dots (on the right) correspond to the cA genes and the remaining genes, respectively. Green lines show orthologous gene relationships defined by BLAST reciprocal best hit.

In prokaryotes, horizontally transferred genes are often clustered in the chromosome and make genomic islands [26,43,44]. We investigated co-localization of the cA genes along the chromosomes of the LDVs using a Monte-Carlo simulation (see **Methods**). None of the genomes of entomopoxviruses, baculoviruses, iridoviruses, mimivirus, nimavirus or phycodnaviruses exhibited a significant propensity for cA genes to be clustered along the genome. Of the 67 LDVs, only 12 presented a significant frequency of pairs of co-localized cA genes (*p *≤ 0.01; Table 1). Using the same Monte-Carlo simulation, we identified significantly large sized cA gene islands (ranging from 3 up to 32 neighboring cA genes) in 11 LDV genomes (*p *≤ 0.01; Additional file 7). Those were 6 betaherpesviruses, 1 alphaherpesvirus, 2 chordopoxviruses, African swine fever virus, and the cyanophage P-SSM4. Betaherpesviruses showing cA islands of significant sizes include murid herpesvirus 2 with an extravagant 53% of cA gene proportion. The abundance of cA gene islands in betaherpesviruses parallels the mosaicity in G+C composition along their genomes [17]. It is known that betaherpesvirus genomes are composed of core genes located in the middle of their genomes and shared by different subfamilies of *Herpesviridae*, plus betaherpesvirus-specific genes located at the extremities of the genomes [45]. As shown in Fig. 5, many of the beta-family genes were identified as cA genes making genomic islands.

#### Correlation between cA genes and functional properties

Functional constraint on specific genomic regions can be a cause of atypical nucleotide compositions of genes. We selected five genomic regions to examine if there were correlations between cA genes and certain of their functional attributes. For the first three cases (gamma- and betaherpseviruses), the correlation between the nucleotide compositional biases and gene functions has already been described, and could serve as a positive control for our method. For the last two cases (*Emiliania huxleyi *virus-86 and mimivirus), no such correlation has been previously reported.

Human herpesvirus 4 (Epstein-Barr virus) of the gammaherpesvirus subfamily has two different life cycle modes: latent and productive. Nine genes are known to be expressed during latency [46]. Those genes are hardly expressed during the productive mode. They are highly A+T rich compared to the remaining genes. Karlin *et al. *hypothesized that the unique codon usage of those genes helps to minimize the competition with host genes for various host resources during latency [19]. We found that eight of the nine latency genes were indeed detected as cA genes (89%). In contrast, the cA gene proportion was significantly lower for the remaining genes (29/81, 36%, *p *= 0.003).

The immediate-early transcription region (≈10-kb) of the murid herpesvirus 1 (murine cytomegalovirus, betaherpesvirus subfamily) genome is known to be CpG suppressed [47]. Experimental data suggest that the methylation of the cytosines in CpG dinucleotides in the enhancer/promoter of these regions has a regulatory role in gene expression [48]. The CpG suppressed region of the murid herpesvirus 1 genome encodes 10 genes. We found that the cA gene proportion in this region (6/10 genes, 60%) was twice as important as in the remaining regions (46/146, 32%) of the genome, though their difference might be due to chance (*p *= 0.085).

The murid herpesvirus 1 genome possesses three (19-kb, 10-kb and 17-kb) regions that are A+T rich relative to other parts of the genome [47]. Those regions are enriched in genes encoding membrane glycoprotein (e.g. m02 and m145 families). Some of those proteins were suggested as responsible for the evasion from natural killer cell-mediated immune surveillance through their interaction with inhibitory natural killer cell receptors [49,50]. Of 37 genes within the A+T rich regions, 67% (24 genes) were detected as cA genes, which is significantly higher than the cA gene proportion for the remaining genes (23.5%, 28/119 genes, *p *< 10^-5^). It is uncertain if the increased A+T levels of these regions have functional roles. However, it should be noted that these A+T rich regions contain five of the seven genes exhibiting significant sequence polymorphisms between strains of murid herpesvirus 1 [51].

*Emiliania huxleyi *virus-86 (EhV-86) of the *Phycodnaviridae *family exhibits two distinct transcription phases during its lytic infection to the host alga, *E. huxleyi *[52]. The primary phase is characterized by the transcriptions of a group of genes by 1 hour post-infection. The secondary phase involves the transcriptions of other genes between 2 and 4 hours post-infection. Respectively thirty-eight and 253 genes in our data set (ORF length ≥ 100 codons) correspond to the primary and the secondary transcription phases. We found a significantly greater fraction of cA genes in the primary phase (8/38, 21%) than in the secondary phase genes (19/253, 7.5%; *p *= 0.014). Genes expressed during the primary phase map in the 104-kb central genomic region (bases 200,000 to 304,000), which shows a similar G+C content as the rest of the genome [53]. It has been suggested that promoter-like elements (family A repeats) uniquely found in this region control this early expression pattern of EhV-86 [7]. The functions of the early expression are unknown as most of the transcribed genes lack detectable homologs in the databases. Allen et al. postulated that an ancestral EhV acquired the 104-kb region by horizontal transfer [53].

Mimivirus has unusually well conserved promoter-like AAAATTGA motifs in the upstream regions of half of its predicted genes in the genome [39]. Based on the putative associated gene functions, Suhre *et al. *predicted that these promoter-like elements regulate gene expression during the early stages of the viral infection, whilst most of the genes contributing to the virus particle are devoid of this motif [54]. Of 402 genes with the promoter-like motifs in our data set, 43 (10.6%) were detected as cA genes. Of 508 genes lacking the motifs, 40 (7.9%) were detected as cA genes. The difference is not statistically significant (*p *= 0.16).

In summary, we found a significant correlation between cA genes and previously described functional categories of genes for three of the five cases. It should be noted that in the case of EhV, no relationship between expression timing and gene nucleotide composition was previously reported. This suggests the possible use of nucleotide composition analysis to predict expression patterns of viral genes. These results indicate that anomalous nucleotide compositions of some of the cA genes can be due to functional constraints, although the distinction between functional constrains and horizontal transfer events is generally difficult given the known bias in functions of horizontally transferred genes in prokaryotes [24,26].

### Many eukaryotic LDVs exhibit cA genes with putative functions associated with host defense systems

We classified the cA genes according to their putative functions based on their annotations and further similarity searches against sequence databases (Additional file 8, Fig. 6). The vast majority of the cA genes have no predicted functions. A notable feature among the remaining cA genes concerns eukaryotic LDVs. We observed that many eukaryotic LDVs possess cA genes putatively associated with the control of host defense systems, such as innate/adaptive immune systems or apoptosis pathways (Additional file 9). Poxviruses, betaherpesviruses and gammaherpesviruses are particularly rich in cA genes of this category (*p *≤ 10^-5^). They present cA genes having host homologs such as cytokine (interleukin), chemokine and MHC class I genes [55]. Remarkably, in nearly all the families of eukaryotic viruses (except for phycodnaviruses), we found species exhibiting cA genes encoding proteins putatively involved in apoptosis pathways (Additional file 9). This suggests a possible central role of apoptotic pathways in eukaryotic LDV-host interactions. Those cA genes may endow these large DNA viruses a capacity to prolong the integrity of the host cells and thereby "buy time" to pursue their replication cycles. Alternatively, viruses may induce host apoptosis to facilitate host cell exit as previously suggested for EhV-86 [7]. The biased nucleotide compositions of cA genes associated with host defense systems may be due to their relatively recent origins (i.e. horizontal gene transfer) and/or particular functional constraints on their nucleotide compositions.

**Figure 6:**
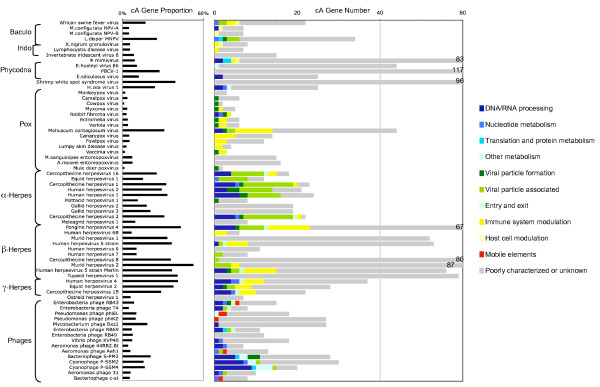
Functional categories of the cA genes detected in the 67 LDV genomes.

We found that the protein product from a cA gene in mimivirus (MIMI_L211 previously annotated as unknown function) exhibits significant sequence similarities to the C-terminal half of the etoposide-induced 24 (EI24) proteins (pfam07264, E-value < 10^-7^). EI24 (also known as PIG8) is directly induced by p53, a critical tumor suppressor coordinating DNA repair, cell-cycle arrest and apoptosis in response to cellular stresses. It has been suggested that EI24 acts as a pro-apoptotic factor and prevents tumor spreading in mammals [56]. The N-terminal part of the EI24, which is missing in MIMI_L211, binds to Bcl-2, while the function of the C-terminus of EI24 is unknown. This is the first identification of an EI24-like domain in a viral genome. The presence of such a domain in mimivirus is puzzling since, to the best of our knowledge, no apoptotic phenomenon has been described for its unicellular host *Acanthamoeba polyphaga*. It is notable that another amoeba *Dictyostelium discoideum*, which carries out apoptotic processes, possesses a hypothetical ORF (Q54PW9_DICDI) matching the EI24 domain (E-value < 10^-19^).

### Phylogenetic evidences for horizontal gene transfers

Horizontally acquired genes may or may not exhibit anomalous nucleotide compositions depending on their origin. Phylogenetic reconstruction is a more powerful approach to examine horizontal gene transfers. However, the approach could be used only for a limited part of our data set, as most of the cA genes have no detectable homologs in the databases. Wherever possible, we systematically conducted phylogenetic reconstructions for the cA genes. Out of a total of 1633 phylogenetic reconstruction, only eight candidate horizontal gene transfer events were supported by the reconstructed trees (using conservative criteria, see **Materials and Methods**). All these trees revealed striking phylogenetic incongruities (Additional file 10): glutathione peroxidase of molluscum contagiosum virus (Additional file 10) (**A**), interleukin 10 of Cercopithecine herpesvirus 15 (**B**), hypothetical protein (R1R) of monkeypox virus (**C**), G protein-coupled receptor of fowlpox virus (**D**), photosystem II D2 protein (**E**) and site-specific DNA methylase (**F**) of bacteriophage S-PM2, thymidylate synthase of cyanophage P-SSM2 (**G**) and cytosine methyl-transferase of cyanophage P-SSM4 (**H**).

## Discussion

We demonstrated that there is no significant correlation between the G+C content of LDV genomes and that of their modern hosts, and that the genomic signatures of LDVs are also significantly different from those of the hosts. Recently, Mrázek and Karlin reported a similar result for large DNA viruses based on a smaller data set than the one used in this study [27]. This simple observation does not favor the "gene pickpockets" depiction of viruses which supposedly frequently acquire genetic material from their hosts [57,58]. Various factors can potentially account for the existence of species-specific global nucleotide compositions [18,22]. Most importantly, LDVs carry their own genes for the major components of replication machinery. Several authors have suggested that viruses are ancient and that their evolutionary trajectory could be largely dissociated from those of their present hosts [2-4]. Accordingly, specific genomic signatures of LDV genomes may originate from intrinsic properties of their replication machinery. As an alternative, we speculate that viruses may take advantage of the compositional differences to re-orient host machineries towards viral DNA/RNA molecules.

We found no significant correlation between the cA gene proportion and the size of the LDV genomes. This observation suggests that extremely large sizes of the genomes of some LDVs such as mimivirus are not due to recent accretion of foreign genes. By extrapolation, the capacity to capture foreign genes is unlikely to be the major factor that determines the tremendous variation in genome size for DNA viruses [6].

We showed that the degree of intra-genome heterogeneity was highly variable across LDVs (i.e. the proportion of cA genes ranging from 1% to 53%). In prokaryotes, anomalous gene nucleotide composition is usually attributed to horizontal gene acquisition events. It has been argued that traces of horizontal gene transfer events as old as 100 million years can be detected through the identification of anomalies in nucleotide composition for bacterial genes [44]. The composition-based method used in this study identified horizontally transferred gene candidates for prokaryotes, which constitute 0% to 25% of their gene complements. Surprisingly the same method identified only a comparable level of cA gene proportions for half of the analyzed LDVs. Furthermore, several LDVs exhibited a cA proportion as low as that of small parasitic intracellular bacteria, rarely exchanging genes with other bacteria. Thus, the nucleotide compositions of LDVs could indicate no general differences between LDVs and prokaryotes in the frequency of horizontal gene acquisitions.

Most of the identified cA genes were of unknown functions and did not exhibit cellular homologs in the databases. This also argues against massive HGTs from hosts to LDVs. One may speculate that cellular homologs could not be detected due to a faster viral sequence evolution. However, a recent study showed that evolutionary rates are similar between LDV genes with database homologs and those lacking detectable homologs (i.e. ORFans) [8].

Furthermore, functional and structural constraints can also cause intra-genome compositional heterogeneity. In this study, we explored several mechanisms that could potentially cause compositional anomaly in LDV genomes. These mechanisms include specific gene functions and replication associated strand asymmetry. We validated a significant enrichment of cA genes in specific functional categories of genes for some of the LDVs including EhV-86. We evidenced the presence of a nucleotide composition bias in the EhV-86 genes expressed in the primary phase that was previously overlooked. In contrast, we showed that replication associated compositional asymmetry is not a major cause of cA gene compositional bias for most of the LDVs.

It should be noted, that highly expressed genes exhibiting biased codon usages are usually pre-excluded in quantifying the number of horizontally transferred genes in prokaryotic genomes [26]. Due to the lack of such a general knowledge on viral codon usage bias with functional consequences, we did not apply such a filter in our computational identification of cA genes. In this regard, possible functional constraints on the nucleotide compositions will tend to contribute to an overestimation of recent horizontal gene transfers in viruses relative to prokaryotes.

The lack of database homologs or phylogenetic evidences does not exclude the possible HGT-origins of cA genes. The hugely diverse world of viruses is probably the most underrepresented in the current database [59]. Thus, we speculate that a significant part of these cA genes might have been transferred from other viruses that are not yet sequenced. Such inter-virus gene exchange can be achieved by illegitimate or homologous recombination [60-62] occasionally leading to the fusion of two viral genomes [63], or with the aid of mobile elements [64] upon co-infection of a cell by multiple virus species. Li *et al. *described a clear case of HGT from *Xestia c-nigrum *granulovirus to *Mamestra configurata *nucleopolyhedrovirus B [65], though this HGT event was not detected by our study due to the similar nucleotide compositions of these two genomes.

Goldenfeld and Woese pointed out the important role of viruses in the gene flux among microbial communities [66]. They conceptualized the viral gene pool as a large dynamic repository of genetic information accessible to microorganisms. Inter-virus gene transfer may be central to the dynamics of the viral gene pool. Given the known diversity of genetic material in the virus world and their under-representation in the current sequence databases [67], this hypothesis appears as plausible as the functional/structural scenario in explaining the existence of compositionally anomalous genes in large DNA viruses.

## Conclusion

The genomes of large DNA viruses exhibit nucleotide compositions largely differing from their host genomes. Our results based on nucleotide composition analyses, database searches and phylogenetic tree reconstructions suggest that horizontal gene transfer to these viruses from their current hosts is infrequent and does not account for the large variation in their genome size. However, the viral genomes still show a variable proportion of compositionally anomalous genes. Such compositional biases potentially arise from particular biological functions at the nucleic acid level and/or inter-virus gene transfers.

## Methods

### Viral genome data

Viral genome data were downloaded from the viral section of the NCBI Reference Sequence (RefSeq) database [68]. We have selected 67 non-redundant double-strand DNA viral genomes larger than 150 kb from the dataset (Table 1).

### Host sequence data

We prepared 32 sets of protein coding sequence (CDS) data for viral hosts (Table S6). For viral hosts species for which complete genome sequences are available, we downloaded sequence data from KEGG [69]. For other viral hosts, we retrieved CDS data from GenBank [70]. We used only CDS longer than or equal to 300 bp. For some of the CDS data sets built from GenBank, we included sequences from organisms that are closely related to the viral host species (Additional file 11). We removed the sequence redundancy in each CDS data set from GenBank by keeping only one representative from a cluster of homologs. Clustering of homologous protein sequences was performed using BLASTCLUST [71]; we used 40% for the minimal sequence identity (-S option) and 75% for the minimal coverage by alignment (-L option) for at least one sequence (-b option). Of the 32 host sequence data sets, 26 sets were composed of more than 20 sequences, and used in this study. These represent the host sequence data for 61 LDV genomes.

### Viral ORF and intergenic sequence data

Based on the RefSeq annotation, we prepared a dataset for CDSs and non-coding sequences (NCDSs) for viral genomes. We retained only CDSs longer than or equal to 300 bp, and discarded shorter ones.

### Genomic signature distance

For the comparison of statistical nucleotide sequence properties of CDSs among viruses and their hosts, we used the method proposed by Sandberg *et al. *[30]. This method defines a genomic signature as a set of frequencies of all overlapping oligonucleotides of specific length *k *(*k *= 4 in this study) in the forward strand of the whole nucleotide sequences available for a given genome. The distance between two genomic signatures (*X *and *Y*) is calculated as an Euclidian distance *D*_E_, using the following formula:

DE(X,Y)=[∑i=1n(Xi−Yi)2]12,
 MathType@MTEF@5@5@+=feaafiart1ev1aaatCvAUfKttLearuWrP9MDH5MBPbIqV92AaeXatLxBI9gBaebbnrfifHhDYfgasaacPC6xNi=xI8qiVKYPFjYdHaVhbbf9v8qqaqFr0xc9vqFj0dXdbba91qpepeI8k8fiI+fsY=rqGqVepae9pg0db9vqaiVgFr0xfr=xfr=xc9adbaqaaeGacaGaaiaabeqaaeqabiWaaaGcbaGaemiraq0aaSbaaSqaaiabdweafbqabaGccqGGOaakcqWGybawcqGGSaalcqWGzbqwcqGGPaqkcqGH9aqpdaWadaqaamaaqahabaWaaeWaaeaacqWGybawdaWgaaWcbaGaemyAaKgabeaakiabgkHiTiabdMfaznaaBaaaleaacqWGPbqAaeqaaaGccaGLOaGaayzkaaWaaWbaaSqabeaacqaIYaGmaaaabaGaemyAaKMaeyypa0JaeGymaedabaGaemOBa4ganiabggHiLdaakiaawUfacaGLDbaadaahaaWcbeqcfayaamaalaaabaGaeGymaedabaGaeGOmaidaaaaakiabcYcaSaaa@4A32@

where *n *= 256 for *k *= 4.

### Identification of compositionally anomalous genes

To detect cA genes in LDV genomes, we used the computer program developed by Nakamura *et al. *[26]. We used a Markov order of 3 with a 96 bp window sliding with a step size of 12 bp along the genome. A Monte-Carlo simulation was used to compute the statistical significance of the nucleotide composition bias in a gene as in Nakamura *et al.*. The threshold for the statistical was set to 1% significance (unilateral statistical test). In addition to the dataset derived from RefSeq, a control dataset was generated from the viral genomic sequences by conserving only open reading frames longer than or equal to 300 bp that exhibit significant sequence similarity hits to database sequences from cellular organisms or distantly related virus, using BLASTP [71] against SwissProt/TrEMBL [72] and the viral RefSeq databases. The proportions of the cA genes for the control dataset varied from 0% to 54% across different viruses, and correlated with the cA gene proportions for the dataset derived from RefSeq (R^2 ^= 0.73, *p *< 0.001, not shown). In the manuscript we show only the results obtained from the RefSeq dataset.

### Statistical test for cA genes co-localization

We conducted two distinct statistical tests for physical proximity of cA genes in a viral genome (i.e. clustering of genes along the chromosome) using a Monte-Carlo simulation. First, we counted the number of pairs (*n*) of cA genes that were consecutively encoded in the genome. We shuffled the order of genes in the genome 1000 times and obtained the distribution of *n *in the randomized data. The distribution was used to determine *p*-value for *n *from the real data. Second, we recorded the size (number of genes, *N*) of each cA gene cluster. From the Monte-Carlo simulation, we obtained the *p*-value for the observation of at least one cA gene cluster with the size equal to or greater than *N *genes.

### Phylogenetic tree reconstruction

All the cA genes were searched against the SwissProt/TrEMBL database using BLASTP (E-value < 10^-5^) to identify homologous sequences. For each gene, we retrieved 50 best hits at the maximum from the database, and aligned them to the candidate sequence using MUSCLE [73]. We improved these initial alignments by removing highly divergent or fragmented sequences with visual inspection of the alignments. The alignments were used to generate neighbor-joining (NJ) trees using CLUSTALW [74] with Kimura's correction for distance. Some of the NJ trees were selected for further analyses by the neighbor-joining method with Jones-Taylor-Thornton (JTT) substitution model [75] using MEGA [76] and the maximum likelihood method by PHYML [77]. The results of the BLAST searches were also served to assign putative functions to the cA genes where the annotations in the RefSeq database were inadequate for our analysis.

### Phylogenetic evidences for HGT

Horizontal gene transfer (HGT) is usually invoked, when a phylogenetic tree based on gene sequences is incongruent with a species tree. In this study, we assumed deep phylogenetic origins for large DNA virus lineages (*i.e. *before the divergence of major phyla of cellular organisms), which have been indicated by the analyses conserved genes (*e.g. *DNA polymerase) [5,78]. To list up candidates of HGT, we first identified cases where the grouping of viral sequences, *V*, and their cellular homologs, *I*, is supported with a high bootstrap value (*i.e. *≥ 80% for (*V*, *I*)]). As the reconstructed tree is unrooted, *V *could represent an outgroup of *I *and other cellular homologs. In this case, HGT between viruses, *V*, and cellular organisms, *I*, is not required to account for the gene tree topology. To eliminate this possibility, we demanded one of the following two additional criteria for potential HGT events.

#### Additional criteria 1

- From independent evidences, one can chose a group of sequences, *O1*, representing an outgroup of *I*.

- From independent evidences, one can chose a group of sequences, *O2*, representing an outgroup of *I *and *O1*. [Consequently, the tree topology will become (*O2*, (*O1*, (*V*, *I*))), when rooted by *O2*.]

#### Additional criteria 2

- There are only a few species and/or genera in *I*.

- Other sequences outside the (*V*, *I*) group are from a wide variety of genera.

- The branches from the root of (*V*, *I*) to *V *and *I *are relatively shorter than the branches from the root of (*V*, *I*) to other peripheral nodes.

## Authors' contributions

AM performed most of the analyses. HO designed most of the experiments and performed part of the analyses. JMC designed part of the experiments. All authors contributed to the writing of the manuscript.

## Supplementary Material

Additional file 1 Genomic G+C content of different groups of LDVs. Click here for file

Additional file 2 Genomic G+C content of the 67 LDVs. Click here for file

Additional file 3 Comparison of the cA gene proportions with the total number of genes encoded in each LDV genomes. Click here for file

Additional file 4 Comparison of the cA gene proportions with genomic G+C content. Click here for file

Additional file 5 Comparison of the nucleotide composition between cA genes and host genes. We used genes from 61 LDV genomes with sufficient amount of host CDS data. For every viral gene, genomic signature distances (i.e. Euclidian distances based on di-nucleotide frequencies) from viral genome (horizontal axis) and from host genome (vertical axis) were computed. (A): non-cA genes. (B) and (C): cA genes. In (C), the vertical axis corresponds to the distance between the cA genes and a randomly chosen host genome. Click here for file

Additional file 6 Sub-strand classification, according to their nucleotide-skew, for 15 LDV genomes. Click here for file

Additional file 7 cA gene clusters of significant size in 11 LDV genomes. Click here for file

Additional file 8 List of the cA genes in the 67 LDV genomes. Click here for file

Additional file 9 Summary of viral genes involved in the control of host defense system. Click here for file

Additional file 10 Phylogenetic trees supporting the HGT events for the cA genes detected by a composition-based approach. (**A**) Glutathione peroxidase, (**B**) Interleukin 10, (**C**) R1R hypothetical protein, (**D**) G protein-coupled receptor, (**E**) Photosystem II D2 protein, (**F**) Site-specific DNA methylase, (**G**) Thymidylate synthase and (**H**) Cytosine methyltransferase. Each node is labeled with SWISS-PROT/TrEMBL entry, the taxonomic group (A for *Archaea*, B for *Bacteria*, E for *Eukaryota *and V for *Virus*) and the specie name. The viral genes detected as cA genes by the method of the Nakamura *et al. *are highlighted in red. "I" corresponds to a clade branching with the viral cA genes; "O1" corresponds to the outgroup of I, and "O2" corresponds to the outgroup of I and O1. Paralogs, orthologs and homologs indicate the relationships between the outgroups (O1 or O2) and the internal groups (I or (I, O1)). (A-E) satisfy the criterion 1, and (F-H) satisfy the criterion 2 (see **Methods**). Tree reconstruction was carried out by the maximum likelihood method. Bootstrap values are indicated as the branch levels. Click here for file

Additional file 11 Nucleotide sequence data for the hosts (or its close relatives) of the 67 LDVs. Click here for file
