# Supplementary material for: Horizontal gene transfer and nucleotide compositional anomaly in large DNA viruses
Source: BMC Genomics. 2007 Dec 10;8:456. doi: 10.1186/1471-2164-8-456 (PMC2211322; doi:10.1186/1471-2164-8-456)
Supplement: Additional file 1 — Genomic G+C content of different groups of LDVs. [file 1471-2164-8-456-S1.pdf]

Genomic G+C content of different groups of LDVs.

**Classification**	**Sub-classification**	**Number of Species**	**Genome Size (kbp)**	**Genomic G+C (%)**
Asfarviridae	*Asfivirus*	1	170	39%
Baculoviridae	*Nucleopolyhedrovirus*	3	155 - 161	40% - 57%
Baculoviridae	*Granulovirus*	1	178	40%
Caudovirales	*Myoviridae*	14	156 - 280	35% - 65%
Caudovirales	*Unclassified*	1	185	26%
Herpesviridae	*Alphaherpesvirinae*	10	150 - 174	44% - 76%
Herpesviridae	*Betaherpesvirinae*	10	153 - 241	36% - 66%
Herpesviridae	*Gammaherpesvirinae*	3	171 - 184	57% - 62%
Herpesviridae	*Unclassified*	1	207	39%
Iridoviridae	*Lymphocystivirus*	1	186	27%
Iridoviridae	*Iridovirus*	1	212	29%
Mimiviridae	*Mimivirus*	1	1181	28%
Nimaviridae	*Whispovirus*	1	305	41%
Phycodnaviridae	*Coccolithovirus*	1	407	40%
Phycodnaviridae	*Chlorovirus*	1	330	40%
Phycodnaviridae	*Phaeovirus*	1	335	52%
Poxviridae	*Chordopoxvirinae*	12	150 - 359	26% - 63%
Poxviridae	*Entomopoxvirinae*	2	232 - 236	18%
Unclassified	*Unclassified*	1	228	42%
