# Supplementary figures and images for: Horizontal gene transfer and nucleotide compositional anomaly in large DNA viruses
Source: BMC Genomics. 2007 Dec 10;8:456. doi: 10.1186/1471-2164-8-456 (PMC2211322; doi:10.1186/1471-2164-8-456)

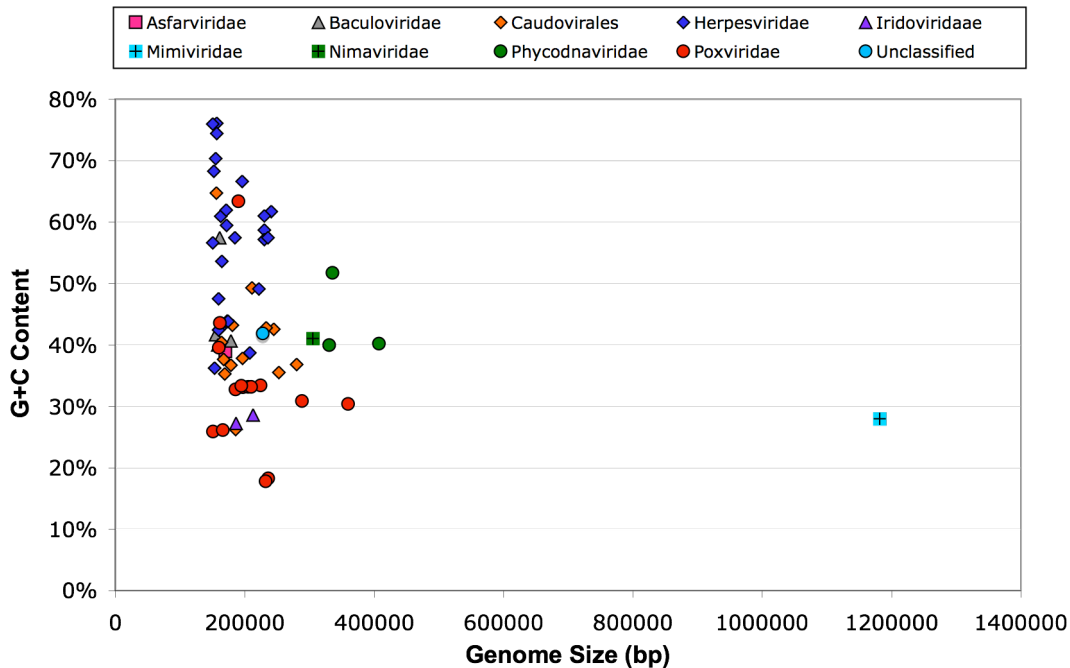

Supplement: Additional file 2 — Genomic G+C content of the 67 LDVs. [file 1471-2164-8-456-S2.pdf]

cA Gene Proportion

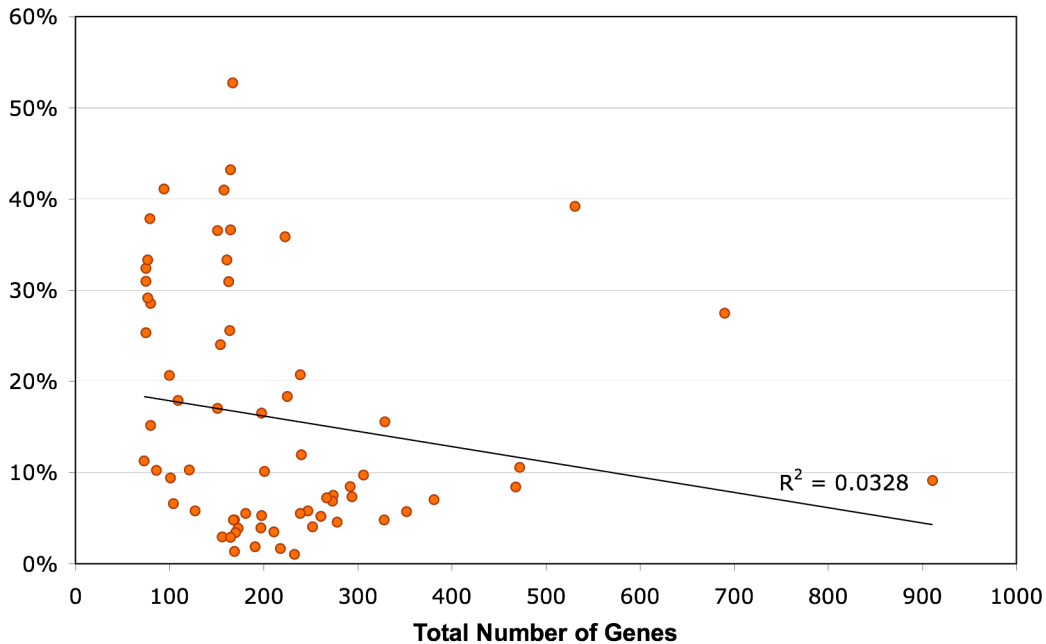

Supplement: Additional file 3 — Comparison of the cA gene proportions with the total number of genes encoded in each LDV genomes. [file 1471-2164-8-456-S3.pdf]

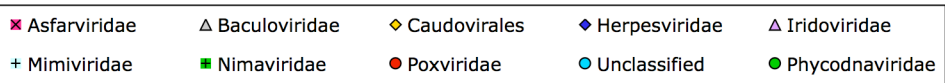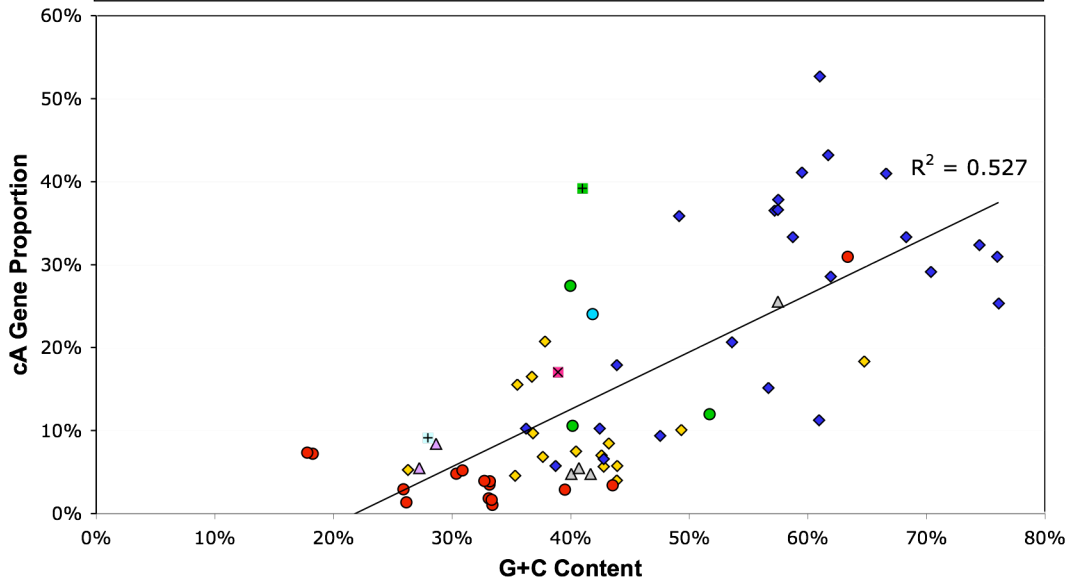

Supplement: Additional file 4 — Comparison of the cA gene proportions with genomic G+C content. [file 1471-2164-8-456-S4.pdf]

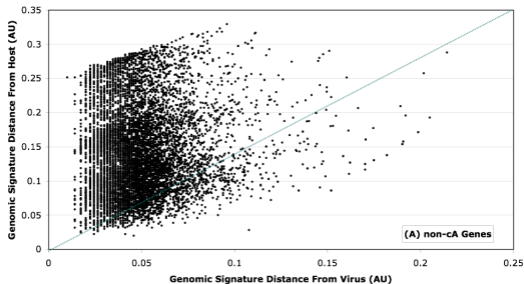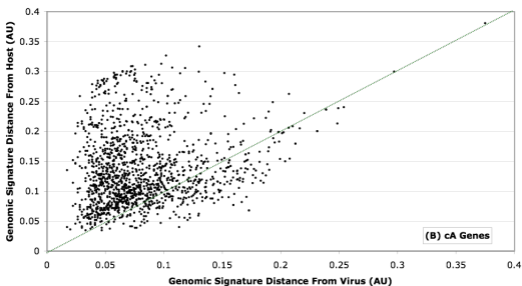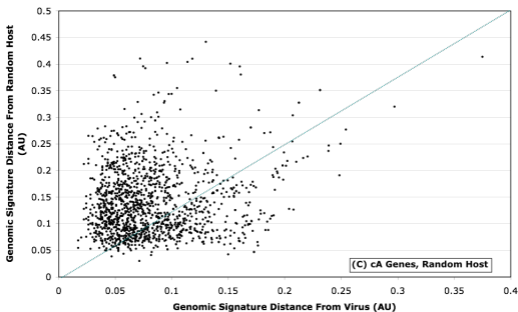

Supplement: Additional file 5 — Comparison of the nucleotide composition between cA genes and host genes. We used genes from 61 LDV genomes with sufficient amount of host CDS data. For every viral gene, genomic signature distances (i.e. Euclidian distances based on di-nucleotide frequencies) from viral genome (horizontal axis) and from host genome (vertical axis) were computed. (A): non-cA genes. (B) and (C): cA genes. In (C), the vertical axis corresponds to the distance between the cA genes and a randomly chosen host genome. [file 1471-2164-8-456-S5.pdf]
