# Supplementary material for: Horizontal gene transfer and nucleotide compositional anomaly in large DNA viruses
Source: BMC Genomics. 2007 Dec 10;8:456. doi: 10.1186/1471-2164-8-456 (PMC2211322; doi:10.1186/1471-2164-8-456)
Supplement: Additional file 6 — Sub-strand classification, according to their nucleotide-skew, for 15 LDV genomes. [file 1471-2164-8-456-S6.pdf]

Sub-strands classification, according to their nucleotide-skew, for 15 LDV genomes.

LDV	Class I segments		Class II segments		Fisher's test
	Position	CDS*	Position	CDS*	*p*-value
**Mycobacteriophage Bxzl**	0..156102	143 (27)	complement(0..156102)	4 (0)	1
***Pseudomonas* phage phiKZ**	0..280334	229 (22)	complement(0..280334)	49 (5)	0.99
**Tupaiid herpesvirus 1**	0..13000, complement(13000..108000), 180000..195859	83 (34)	complement(0..13000), 13000..108000, complement(180000..195859)	61 (25)	1
**Human herpesvirus 5 strain AD169**	complement(0..93000), 93000..190000, complement(190000..230287)	73 (19)	0..93000, complement(93000..190000), 190000..230287	72 (34)	0.0098
**Human herpesvirus 5 strain merlin**	complement(0..93000), 93000..200000, complement(200000..235645)	71 (17)	0..93000, complement(93000..200000), 200000..235645)	82 (39)	0.002
**Human herpesvirus 6(A)**	complement(0..68000), 68000..135000, complement(135000..159321)	61 (4)	0..68000, complement(68000..135000), 135000..159321	46 (7)	0.2
**Human herpesvirus 6B**	complement(0..68000), 68000..1400000, complement(140000..162114)	54 (4)	0..68000, complement(68000.140000), 140000..162114	37 (2)	1
**Human herpesvirus 7**	complement(0..62000), 62000..140000, complement(140000..153080)	43 (2)	0..62000, complement(62000..140000), 140000..143080	35 (6)	0.13
**Fowlpox virus**	0..125000, complement(125000..288539)	71 (7)	complement(0..125000), 125000..288539	159 (5)	0.05
**Canarypox virus**	0..175000, complement(175000..359853)	93 (8)	complement(0..175000), 175000..359853	198 (6)	0.072
***A.moorei* entomopoxvirus**	0..232392	94 (7)	complement(0..232392)	124 (9)	0.99
**Invertebrate iridescent virus 6**	0..45000, complement(45000..113000), 113000..212482	74 (7)	complement(0..45000), 45000..113000, complement(113000..212482)	104 (8)	0.78
**Shrimp white spot syndrome virus**	0..305107	133 (47)	complement(0..305107)	112 (49)	0.19
**African swine fever virus**	complement(0..74000), 74000..161000, complement(161000..170101)	56 (9)	0..74000, complement(74000..161000), 161000..170101	73 (13)	0.81
***A.polyphaga* mimivirus**	0..380000, complement(380000..1181404)	333 (28)	complement(0..380000), 380000..1181404	577 (55)	0.633

* Number of CDS analysed in this study (*i.e.* length ≥ 100 amino-acids). Numbers of identified cA genes are shown in parentheses.
