# Supplementary material for: Horizontal gene transfer and nucleotide compositional anomaly in large DNA viruses
Source: BMC Genomics. 2007 Dec 10;8:456. doi: 10.1186/1471-2164-8-456 (PMC2211322; doi:10.1186/1471-2164-8-456)
Supplement: Additional file 7 — cA gene clusters of significant size in 11 LDV genomes. [file 1471-2164-8-456-S7.pdf]

cA gene clusters of significant size in 11 LDV genomes.

LDV		Protein ID	Annotation	Statistical Test
**Human herpesvirus 5 strain AD169**		NP_899637	Ig Fc-binding glycoprotein RL11	$p \leq 0.001$
		NP_899638	Ig Fc-binding glycoprotein RL12	
		NP_899639	unknown	
		NP_039934	unknown	
		NP_039935	unknown	
		NP_039938	unknown	
		NP_039939	unknown	
		NP_039940	unknown	
		NP_039941	unknown	
		NP_039942	unknown	
		NP_039943	unknown	
		NP_783772	unknown	
		NP_039945	Membrane protein precursor	
		NP_039947	unknown	
**Molluscum contagiosum virus**		NP_044106	Putative non-globular, membrane associated protein	$p \leq 0.009$
		NP_044108	Putative membrane receptor	
		NP_044109	unknown	
		NP_044110	Flice-like protein (apoptosis regulator)	
		NP_044111	Flice-like protein (apoptosis regulator)	
		NP_044112	Slam-like protein	
		NP_044113	Slam-like protein	
		NP_044114	unknown	
**Meleagrid herpesvirus 1**		NP_073361	unknown	$p \leq 0.003$
		NP_073362	unknown w/ Pro-Asn repeat	
		NP_073363	unknown	
		NP_073364	unknown	
**Murid herpesvirus 2**	**cluster 1**	NP_064217	Glycoprotein L precursor	$p \leq 0.001$
		NP_064218	Mucin (fragment)	
		NP_064219	unknown	
		NP_064220	unknown	
		NP_064221	unknown	
		NP_064222	unknown	
		NP_064223	unknown	
		NP_064224	unknown	
		NP_064225	unknown	
		NP_064226	unknown	
		NP_064227	unknown	
		NP_064228	unknown	
		NP_064229	Large tegument protein	
		NP_064230	unknown	
		NP_064231	unknown	
		NP_064232	unknown	
		NP_064233	unknown	
		NP_064234	unknown	
		NP_064235	unknown	
		NP_064236	unknown	
		NP_064237	unknown	
		NP_064238	Non-structural protein NS	
		NP_064239	Immediate-early protein 2 (IE2)	
		NP_064240	CC chemokine homolog	
		NP_064241	unknown	
	**cluster 2**	NP_064243	unknown	$p \leq 0.001$
		NP_064244	unknown	
		NP_064245	unknown	
		NP_064246	unknown	
		NP_064247	Tegument protein	
		NP_064248	Tegument protein	
		NP_064249	Tegument protein	
		NP_064250	unknown	
		NP_064251	MHC class Ib antigen	
		NP_064252	unknown	
		NP_064253	unknown	
		NP_064254	unknown	
		NP_064255	unknown	
		NP_064256	unknown	
		NP_064257	unknown	
		NP_064258	unknown	
		NP_064259	unknown	
		NP_064260	unknown	
		NP_064261	unknown	
		NP_064262	unknown	
		NP_064263	unknown	
		NP_064264	unknown	
		NP_064265	unknown	
		NP_064266	unknown	
		NP_064267	unknown	
		NP_064268	unknown	
		NP_064269	unknown	
		NP_064270	unknown	
		NP_064271	unknown	
		NP_064272	unknown	
		NP_064273	unknown	
		NP_064274	unknown	

LDV	Protein ID	Annotation	Statistical Test
**Tupaiid herpesvirus 1**	NP_116344	unknown	$p \leq 0.001$
	NP_116346	unknown	
	NP_116347	unknown	
	NP_116348	unknown	
	NP_116349	unknown	
	NP_116351	unknown	
	NP_116352	unknown	
	NP_116353	unknown	
	NP_116354	unknown	
	NP_116355	unknown	
	NP_116356	unknown	
	NP_116358	unknown	
	NP_116359	unknown	
	NP_116360	unknown	
	NP_116361	unknown	
	NP_116362	unknown	
	NP_116363	unknown	
	NP_116364	unknown	
**Pongine herpesvirus 4** cluster 1	NP_612643	unknown	$p \leq 0.001$
	NP_612644	Virion glycoprotein RL10	
	NP_612645	Ig Fc-binding glycoprotein RL11	
	NP_612646	Glycoprotein RL12	
	NP_612647	Glycoprotein TRL13	
	NP_612649	unknown	
	NP_612650	unknown	
	NP_612651	Glycoprotein UL6	
	NP_612652	Glycoprotein UL7	
	NP_612653	Glycoprotein UL8	
	NP_612654	Glycoprotein UL9	
	NP_612655	Glycoprotein UL10	
	NP_612656	Glycoprotein UL11	
	NP_612657	unknown	
**Pongine herpesvirus 4** cluster 2	NP_612762	Glycoprotein UL139	$p \leq 0.009$
	NP_612763	unknown	
	NP_612764	unknown	
	NP_612765	unknown	
	NP_612766	unknown	
	NP_612771	unknown	
	NP_612772	unknown	
	NP_612773	unknown	
	NP_612774	unknown	
	NP_612775	Transcriptional transactivator TRS1	
	NP_612776	unknown	
**Human herpesvirus 5 strain merlin**	YP_081459	IgG Fc-binding glycoprotein RL11	$p \leq 0.001$
	YP_081460	IgG Fc-binding glycoprotein RL12	
	YP_081461	Glycoprotein RL11 family member	
	YP_081462	Glycoprotein RL11 family member	
	YP_081464	unknown	
	YP_081465	unknown	
	YP_081466	Glycoprotein RL11 family member	
	YP_081467	Glycoprotein RL11 family member	
	YP_081468	Glycoprotein RL11 family member	
	YP_081469	Glycoprotein RL11 family member	
	YP_081470	Glycoprotein RL11 family member	
	YP_081471	Glycoprotein RL11 family member	
	YP_081472	unknown	
**African swine fever virus**	NP_042702	unknown	$p \leq 0.008$
	NP_042703	unknown	
	NP_042704	Structural protein p22	
	NP_042706	unknown	
	NP_042707	unknown	
**Murid herpesvirus 1**	YP_214016	Glycoprotein family m02	$p \leq 0.001$
	YP_214017	Glycoprotein family m02	
	YP_214018	Glycoprotein family m02	
	YP_214019	Glycoprotein family m02	
	YP_214020	Glycoprotein family m02	
	YP_214021	Glycoprotein family m02	
	YP_214022	Glycoprotein family m02	
	YP_214023	Glycoprotein family m02	
	YP_214024	Glycoprotein family m02	
**Camelpox**	NP_570395	unknown	$p \leq 0.001$
	NP_570396	T1R protein	
	NP_570398	unknown	
**Cyanophage P-SSM4**	YP_214677	RNA-DNA / DNA-DNA helicase ATPase, UvsW	$p \leq 0.009$
	YP_214678	unknown	
	YP_214679	gp55 T4-like sigma factor	
	YP_214680	gp47 T4-like endonuclease	
	YP_214682	gp46 T4-like endonuclease	
