# Supplementary material for: Horizontal gene transfer and nucleotide compositional anomaly in large DNA viruses
Source: BMC Genomics. 2007 Dec 10;8:456. doi: 10.1186/1471-2164-8-456 (PMC2211322; doi:10.1186/1471-2164-8-456)
Supplement: Additional file 8 — List of the cA genes in the 67 LDV genomes. [file 1471-2164-8-456-S8.pdf]

List of the cA genes in the 67 LDV genomes

Classification	Species	Genome ID	Protein ID	Annotation	Functional Category
Asfarvirus					
	African swine fever virus	NC_001659.1	NP_042704.1	Structural protein p22	Viral particle associated
			NP_042727.1	IAP homolog	Immune system modulation
			NP_042729.1	Thymidylate kinase	Nucleotide metabolism
			NP_042751.1	Lectin-like protein	Immune system modulation
			NP_042752.1	CD2 homolog	Host cell modulation
			NP_042818.1	Structural protein p54	Viral particle associated
				Genes of unknown function: 16	
Baculoviridae					
	Mamestra configurata NPV-A	NC_003529.1	NP_613130.1	Actin-rearrangement-inducing factor	Host cell modulation
			NP_613232.1	Late expression factor 11 (LEF-11)	DNA/RNA processing
				Genes of unknown function: 5	
	Mamestra configurata NPV-B	NC_004117.1	NP_689217.1	Actin-rearrangement-inducing factor	Host cell modulation
				Genes of unknown function: 6	
	Lymantria dispar MNPV	NC_001973.1	NP_047638.1	Capsid associated protein	Viral particle associated
			NP_047640.1	Mucin-like protein	Poorly characterized
			NP_047668.1	Ld-bro-a	Poorly characterized
			NP_047674.1	Late expression factor 6	Poorly characterized
			NP_047679.1	Viral ubiquitin	Translation and protein metabolism
			NP_047681.1	Late expression factor 11	Poorly characterized
			NP_047700.1	Few polyhedra protein	Viral particle formation
			NP_047727.1	Telokin-like protein-20	Poorly characterized
			NP_047742.1	Capsid protein P80	Viral particle associated
			NP_047753.1	dUTPase	Nucleotide metabolism
			NP_047768.1	Occlusion-derived virus envelope protein E66 (ODV-E66)	Viral particle associated
			NP_047783.1	Ld-bro-l	Poorly characterized
			NP_047790.1	Ld-bro-n	Poorly characterized
			NP_047791.1	Ld-bro-o	Poorly characterized
				Genes of unknown function: 20	
	Xestia c-nigrum granulovirus	NC_002331.1	NP_059233.1	Fibroblast growth factor 1	Host cell modulation
			NP_059285.1	Putative inhibitor of apoptosis protein	Immune system modulation
				Genes of unknown function: 6	
Iridovirus					
	Lymphocystis disease virus	NC_005902.1	YP_073512.1	Caspase-1 precursor	Immune system modulation
				Genes of unknown function: 6	
	Invertebrate iridescent virus 6	NC_003038.1		Genes of unknown function: 15	
Mimivirus					
	Acanthamoeba polyphaga mimivirus	NC_006450.1	YP_142435.1	Ankyrin containing protein	Poorly characterized
			YP_142449.1	Ankyrin containing protein	Poorly characterized
			YP_142462.1	PCNA	DNA/RNA processing
			YP_142522.1	F-BOX domain	Translation and protein metabolism
			YP_142565.1	Etoposide-induced protein 24 (p53-induced protein 8)	Immune system modulation
			YP_142586.1	Serine/threonine protein kinase	Poorly characterized
			YP_142654.1	Endo/excinuclease amino terminal domain	DNA/RNA processing
			YP_142665.1	RING domain	Poorly characterized
			YP_142797.1	Thioredoxin	Translation and protein metabolism
			YP_142877.1	Rhomboid family protein	Host cell modulation
			YP_142956.1	Ankyrin containing protein	Poorly characterized
				Genes of unknown function: 72	

Classification	Species	Genome ID	Protein ID	Annotation	Functional Category
Phycodnavirus					
	Emiliania huxleyi virus 86	NC_007346.1	YP_293786.1	Putative membrane protein	Poorly characterized
			YP_293842.1	Putative membrane protein	Poorly characterized
			YP_293845.1	Putative membrane protein	Poorly characterized
			YP_293870.1	Putative membrane protein	Poorly characterized
			YP_293930.1	Putative membrane protein	Poorly characterized
			YP_294074.1	Putative membrane protein	Poorly characterized
			YP_294173.1	Putative fatty acid desaturase	Other metabolism
				Genes of unknown function: 37	
	Paramecium bursaria Chlorella virus 1			Genes of unknown function: 117	
	Ectocarpus siliculosus virus 1	NC_002687.1	NP_077501.1	Endonuclease	DNA/RNA processing
			NP_077571.1	Transmembrane protein A	Poorly characterized
			NP_077709.1	Replication factor C3	DNA/RNA processing
				Genes of unknown function: 22	
Nimavirus					
	Shrimp white spot syndrome virus			Genes of unknown function: 96	
Unclassified virus					
	Heliothis zea virus 1	NC_004156.1	NP_690426.1	Dihydrofolate reductase	Nucleotide metabolism
			NP_690455.1	DNA ligase III	DNA/RNA processing
			NP_690456.1	FtsJ-like methyltransferase	DNA/RNA processing
			NP_690540.1	Very late factor 1 VLF-1	Poorly characterized
			NP_690564.1	Carboxylesterase	Other metabolism
				Genes of unknown function: 20	
Chordopoxvirus					
	Monkeypox virus	NC_003310.1	NP_536611.1	T1R protein	Poorly characterized
				Genes of unknown function: 2	
	Camelpox virus	NC_003391.1	NP_570396.1	T1R protein	Poorly characterized
			NP_570516.1	Putative IMV membrane morphogenesis protein	Viral particle formation
				Genes of unknown function: 4	
	Cowpox virus	NC_003663.2	NP_619933.1	Putative phosphorylated IMV membrane protein	Viral particle formation
				Genes of unknown function: 1	
	Myxoma virus	NC_001132.2	NP_051821.1	Putative phosphorylated IMV membrane protein	Viral particle formation
			NP_051855.1	Ig domain OX-2-like protein	Immune system modulation
				Genes of unknown function: 3	
	Rabbit fibroma virus	NC_001266.1	NP_051901.1	dUTPase	Nucleotide metabolism
			NP_051974.1	MutT (NUDIX) protein	DNA/RNA processing
			NP_051996.1	Putative phosphorylated IMV membrane protein	Viral particle formation
			NP_052027.1	Ig domain OX-2-like protein	Immune system modulation
				Genes of unknown function: 0	
	Ectromelia virus	NC_004105.1	NP_671525.1	C-type lectin-like protein	Immune system modulation
			NP_671639.1	Putative phosphorylated IMV membrane protein	Viral particle formation
			NP_671657.1	Leukocyte surface antigen CD47 precursor (Integrin-associated protein) (IAP)	Immune system modulation
			NP_671661.1	Putative type-I membrane glycoprotein	Poorly characterized
				Genes of unknown function: 2	

Classification	Species	Genome ID	Protein ID	Annotation	Functional Category
	Variola virus	NC_001611.1	NP_042165.1	Putative phosphorylated IMV membrane protein	Viral particle formation
			NP_042191.1	Leukocyte surface antigen CD47 precursor (Integrin-associated protein) (IAP)	Immune system modulation
			NP_042194.1	Semaphorin	Immune system modulation
			NP_042198.1	Putative type-I membrane glycoprotein	Poorly characterized
			NP_042218.1	Ankyrin repeat protein	Poorly characterized
				Genes of unknown function: 1	
	Molluscum contagiosum virus	NC_001731.1	NP_043957.1	Contains large predicted non-globular regions and Q/A repeats	Poorly characterized
			NP_043969.1	Poxvirus F11 protein, an early protein.	Poorly characterized
			NP_044002.1	Contains a predicted signal peptide and an immunoglobulin domain	Poorly characterized
			NP_044004.1	Interleukin-18-binding protein precursor (IL-18BP) (Tadekinig-alfa).	Immune system modulation
			NP_044005.1	Interleukin-18-binding protein precursor (IL-18BP) (Tadekinig-alfa).	Immune system modulation
			NP_044017.2	Glutathione peroxidase	Immune system modulation
			NP_044031.1	MHC class I homolog	Immune system modulation
			NP_044074.1	DEAD-like helicases superfamily	DNA/RNA processing
			NP_044078.1	Holliday junction resolvase	DNA/RNA processing
			NP_044082.1	A type inclusion protein	Viral particle formation
			NP_044093.1	Putative membrane protein	Poorly characterized
			NP_044094.1	Putative extracellular virion glycoprotein	Viral particle associated
			NP_044097.1	Putative extracellular enveloped virion protein	Viral particle associated
			NP_044099.1	C-C chemokine-like protein	Immune system modulation
			NP_044106.1	Putative non-globular, membrane-associated protein	Poorly characterized
			NP_044108.1	Putative membrane receptor	Poorly characterized
			NP_044110.1	DED domain; CASP8 and FADD-like apoptosis regulator (FLICE-like protein)	Immune system modulation
			NP_044111.1	DED domain; CASP8 and FADD-like apoptosis regulator (FLICE-like protein)	Immune system modulation
			NP_044112.1	SLAM-like protein	Immune system modulation
			NP_044113.1	SLAM-like protein	Immune system modulation
				Genes of unknown function: 24	
	Canarypox virus	NC_005309.1	NP_955026.1	C-type lectin-like protein	Immune system modulation
			NP_955062.1	Chemokine-like receptor 1	Immune system modulation
			NP_955068.1	Chemokine-like receptor 1	Immune system modulation
			NP_955338.1	Chemokine receptor-like 1	Immune system modulation
			NP_955349.1	C-type lectin-like protein	Immune system modulation
				Genes of unknown function: 9	
	Fowlpox virus	NC_002188.1	NP_038984.1	C5a anaphylatoxin chemotactic receptor (C5a-R) (C5aR) (CD88 antigen).	Immune system modulation
			NP_038990.1	Chemokine receptor-like	Immune system modulation
			NP_039174.1	C-type lectin-like protein	Immune system modulation
				Genes of unknown function: 9	
	Lumpy skin disease virus	NC_003027.1	NP_150435.1	Modulator of immune recognition 1 homolog	Immune system modulation
			NP_150444.1	RING-variant domain	Poorly characterized
			NP_150562.1	Leukocyte surface antigen CD47 precursor (Integrin-associated protein) (IAP)	Immune system modulation
				Genes of unknown function: 1	

Classification	Species	Genome ID	Protein ID	Annotation	Functional Category
	Vaccinia virus	NC_006998.1	YP_233019.1	Putative phosphorylated IMV membrane protein	Viral particle formation
			YP_233046.1	Semaphorin-like protein	Immune system modulation
			YP_233061.1	Secreted TNF-receptor-like protein	Immune system modulation
				Genes of unknown function: 0	
Entomopoxvirus					
	Melanoplus sanguinipes entomopoxvirus	NC_001993.1		Genes of unknown function: 15	
	Amsacta moorei entomopoxvirus 'L'	NC_002520.1	NP_064975.1	Protein phosphatase 1, regulatory subunit 15A	Poorly characterized
			NP_065005.1	Similar to wingless protein receptor Cfz2	Poorly characterized
				Genes of unknown function: 14	
Unclassified Poxvirus					
	Mule deer poxvirus	NC_006966.1	YP_227493.1	Putative phosphorylated IMV membrane protein	Viral particle formation
				Genes of unknown function: 1	
Alphaherpesvirinae					
	Cercopithecine herpesvirus 16	NC_007653.1	YP_443846.1	Trans-acting transcriptional protein ICP0	DNA/RNA processing
			YP_443847.1	Virion membrane glycoprotein L	Viral particle associated
			YP_443859.1	Probable serine/threonine-protein kinase	Viral particle associated
			YP_443860.1	Minor tegument protein	Viral particle associated
			YP_443870.1	Nonglycosylated membrane-associated protein	Poorly characterized
			YP_443883.1	Very large tegument protein	Viral particle associated
			YP_443896.1	Capsid/tegument-associated phosphoprotein	Viral particle associated
			YP_443899.1	Capsid/tegument-associated phosphoprotein	Viral particle associated
			YP_443902.1	Immediate-early protein ICP27 (transcriptional activator)	DNA/RNA processing
			YP_443904.1	Type II membrane protein; involved in virus pathogenicity	Poorly characterized
			YP_443905.1	Immediate-early protein ICP0	DNA/RNA processing
			YP_443907.1	Immediate-early protein ICP22	DNA/RNA processing
			YP_443908.1	Tegument protein	Viral particle associated
			YP_443910.1	Virion membrane glycoprotein G; entry into polarized cells	Entry and exit
			YP_443911.1	Glycoprotein J blocking apoptosis	Immune system modulation
			YP_443915.1	Nucleolar phosphoprotein	Poorly characterized
			YP_443917.1	Tegument protein	Viral particle associated
			YP_443918.1	RNA-binding tegument protein interacting with PKR	Immune system modulation
				Genes of unknown function: 0	
	Equid herpesvirus 1	NC_001491.2	YP_053054.1	dUTPase	Nucleotide metabolism
			YP_053062.1	Virion membrane protein	Viral particle associated
			YP_053083.1	Virion protein	Viral particle associated
			YP_053094.1	Virion protein kinase (serine/threonine protein kinase)	Viral particle associated
			YP_053106.1	Envelop glycoprotein L	Viral particle associated
			YP_053112.1	Glycoprotein	Poorly characterized
			YP_053115.1	Envelope glycoprotein	Viral particle associated
			YP_053116.1	Envelope glycoprotein	Viral particle associated
			YP_053117.1	Envelope glycoprotein	Viral particle associated
				Genes of unknown function: 3	

Classification	Species	Genome ID	Protein ID	Annotation	Functional Category
	Cercopithecine herpesvirus 1	NC_004812.1	NP_851859.1	Trans-acting transcriptional protein ICP0	DNA/RNA processing
			NP_851860.1	Virion glycoprotein L	Viral particle associated
			NP_851872.1	Virion protein kinase	Viral particle associated
			NP_851873.1	Minor tegument protein	Viral particle associated
			NP_851883.1	Virion associated membrane prptein	Viral particle associated
			NP_851886.1	Scaffolding protein	Viral particle formation
			NP_851896.1	Very large tegument protein	Viral particle associated
			NP_851898.1	Capsid protein VP19C	Viral particle associated
			NP_851902.1	DNA polymerase subunit	DNA/RNA processing
			NP_851903.1	Membrane-associated protein	Poorly characterized
			NP_851909.1	Major tegument protein VP22	Viral particle associated
			NP_851911.1	dUTPase	Nucleotide metabolism
			NP_851912.1	Capsid/tegument-associated phosphoprotein	Viral particle associated
			NP_851915.1	Immediate-early protein ICP27 (transcriptional activator)	DNA/RNA processing
			NP_851917.1	Type II membrane protein	Poorly characterized
			NP_851918.1	Trans-acting transcriptional protein ICP0	DNA/RNA processing
			NP_851920.1	Immediate-early protein ICP22	DNA/RNA processing
			NP_851921.1	Tegument protein	Viral particle associated
			NP_851923.1	Virion glycoprotein G	Viral particle associated
			NP_851926.1	Virion glycoprotein I	Viral particle associated
			NP_851928.1	Nucleolar phosphoprotein	Poorly characterized
			NP_851930.1	Tegument protein	Viral particle associated
			NP_851931.1	RNA-binding tegument protein interacting with PKR	Immune system modulation
				Genes of unknown function: 0	
	Human herpesvirus 2	NC_001798.1	NP_044469.1	Trans-acting transcriptional protein ICP0	DNA/RNA processing
			NP_044483.1	Minor tegument protein	Viral particle associated
			NP_044493.1	Nonglycosylated membrane-associated protein	Viral particle associated
			NP_044495.1	Capsid maturation protease	Viral particle formation
			NP_044496.1	Capsid maturation protease	Viral particle formation
			NP_044515.1	Tegument/envelope protein	Viral particle associated
			NP_044522.1	Capsid/tegument-associated phosphoprotein	Viral particle associated
			NP_044525.1	Immediate-early protein ICP27 (transcriptional activator)	DNA/RNA processing
			NP_044526.1	Nuclear matrix-associated protein	Viral particle formation
			NP_044528.1	Trans-acting transcriptional protein ICP0	DNA/RNA processing
			NP_044534.1	Virion glycoprotein G	Viral particle associated
			NP_044537.1	Virion glycoprotein I	Viral particle associated
			NP_044538.1	Virion glycoprotein E	Viral particle associated
			NP_044542.1	Virion protein (RNA-binding)	Viral particle associated
				Genes of unknown function: 7	
	Human herpesvirus 1	NC_001806.1	NP_044600.1	Neurovirulence factor (ICP34.5)	DNA/RNA processing
			NP_044601.1	Trans-acting transcriptional protein ICP0	DNA/RNA processing
			NP_044602.1	Virion glycoprotein L	Viral particle associated
			NP_044603.1	Uracil-DNA glycosylase	DNA/RNA processing
			NP_044604.1	Nuclear phosphoprotein UL3	Poorly characterized
			NP_044627.1	Capsid maturation protease	Viral particle formation
			NP_044628.1	Capsid maturation protease	Viral particle formation
			NP_044636.1	Virion associated phosphoprotein	Viral particle associated
			NP_044647.1	Tegument/envelope protein	Viral particle associated
			NP_044651.1	Tegument protein	Viral particle associated
			NP_044657.1	Immediate early protein	Poorly characterized
			NP_044659.1	Type II membrane protein UL56	Poorly characterized

Classification	Species	Genome ID	Protein ID	Annotation	Functional Category
	Human herpesvirus1		NP_044660.1	Trans-acting transcriptional protein ICP0	DNA/RNA processing
			NP_044661.1	Neurovirulence factor (ICP34.5)	DNA/RNA processing
			NP_044663.1	Immediate early protein ICP22	DNA/RNA processing
			NP_044666.1	Virion glycoprotein G	Viral particle associated
			NP_044670.1	Virion glycoprotein E	Viral particle associated
			NP_044671.1	Nucleolar phosphoprotein	Poorly characterized
			NP_044673.1	Virion protein	Viral particle associated
			NP_044674.1	Virion protein (RNA-binding)	Viral particle associated
				Genes of unknown function: 4	
	Psittacid herpesvirus 1	NC_005264.1	NP_944388.1	Membrane protein	Poorly characterized
			NP_944398.1	Capsid maturation protease	Viral particle formation
			NP_944449.1	glycoprotein I	Poorly characterized
				Genes of unknown function: 5	
	Gallid herpesvirus 2	NC_002229.2	NP_057741.1	Arg-rich protein	Poorly characterized
			NP_057748.1	PCNB like protein	Poorly characterized
			NP_057828.1	Marek's disease gammaherpesvirus tumorigenicity associated mRNA	Poorly characterized
			NP_057749.1	Marek's disease gammaherpesvirus tumorigenicity associated mRNA	Poorly characterized
			NP_057829.1	PCNB like protein	Poorly characterized
			NP_057836.1	Arg-rich protein	Poorly characterized
				Genes of unknown function: 13	
	Gallid herpesvirus 3	NC_002577.1		Genes of unknown function: 19	
	Cercopithecine herpesvirus 2	NC_006560.1	YP_164442.1	Trans-acting transcriptional protein ICP0	DNA/RNA processing
			YP_164443.1	Virion glycoprotein L	Viral particle associated
			YP_164444.1	Uracil-DNA glycosylase	DNA/RNA processing
			YP_164455.1	Virion protein kinase	Viral particle associated
			YP_164456.1	Minor tegument protein	Viral particle associated
			YP_164466.1	Nonglycosylated virion membrane-associated protein	Viral particle associated
			YP_164469.1	Scaffolding protein	Viral particle formation
			YP_164479.1	Very large tegument protein	Viral particle associated
			YP_164480.1	Minor tegument protein	Viral particle associated
			YP_164492.1	Major tegument protein VP22	Viral particle associated
			YP_164494.1	dUTPase	Nucleotide metabolism
			YP_164495.1	Capsid/tegument-associated phosphoprotein	Viral particle associated
			YP_164498.1	Immediate-early protein ICP27 (transcriptional activator)	DNA/RNA processing
			YP_164500.1	Type II membrane protein	Poorly characterized
			YP_164501.1	Trans-acting transcriptional protein ICP0	DNA/RNA processing
			YP_164503.1	Immediate-early protein ICP22	DNA/RNA processing
			YP_164504.1	Tegument protein	Viral particle associated
			YP_164506.1	Virion glycoprotein G, involved in entry into polarized cells	Entry and exit
			YP_164509.1	Virion glycoprotein I	Viral particle associated
			YP_164510.1	Virion glycoprotein E	Viral particle associated
			YP_164513.1	Tegument protein	Viral particle associated
			YP_164514.1	RNA-binding tegument protein interacting with PKR	Immune system modulation
				Genes of unknown function: 0	
	Meleagrid herpesvirus 1	NC_002641.1		Genes of unknown function: 8	

Classification	Species	Genome ID	Protein ID	Annotation	Functional Category
Betaherpesvirus					
	Pongine herpesvirus 4	NC_003521.1	NP_612644.1	Virion glycoprotein RL10	Viral particle associated
			NP_612645.1	IgG Fc-binding glycoprotein RL11	Immune system modulation
			NP_612646.1	Glycoprotein RL12	Poorly characterized
			NP_612647.1	Glycoprotein TRL13	Poorly characterized
			NP_612651.1	Glycoprotein UL6	Poorly characterized
			NP_612652.1	Glycoprotein UL7	Poorly characterized
			NP_612653.1	Glycoprotein UL8	Poorly characterized
			NP_612654.1	Glycoprotein UL9	Poorly characterized
			NP_612655.1	Glycoprotein UL10	Poorly characterized
			NP_612656.1	Glycoprotein UL11	Poorly characterized
			NP_612662.1	MHC class I-like protein UL18	Immune system modulation
			NP_612664.1	Glycoprotein UL20	Poorly characterized
			NP_612666.1	Glycoprotein UL22A	Poorly characterized
			NP_612676.1	pp150 tegument protein UL3	Viral particle associated
			NP_612681.1	Viral mitochondrial inhibitor of apoptosis (vMIA)	Immune system modulation
			NP_612694.1	Membrane-associated protein UL50 involved in egress of capsids from nucleus	Entry and exit
			NP_612695.1	DNA packaging protein UL51	Viral particle formation
			NP_612706.1	Envelope glycoprotein UL73	Viral particle associated
			NP_612707.1	Glycoprotein UL74	Poorly characterized
			NP_612721.1	Virion protein UL88	Viral particle associated
			NP_612727.1	Tegument protein UL95	Viral particle associated
			NP_612731.1	Myristylated tegument protein UL99	Viral particle associated
			NP_612737.1	UL112 involved in recruiting DNA replication proteins to nuclear replication compartments	DNA/RNA processing
			NP_612743.1	Glycoprotein UL120	Poorly characterized
			NP_612744.1	Membrane protein UL121	Poorly characterized
			NP_612745.1	Immediate-early transcriptional regulator UL122	DNA/RNA processing
			NP_612746.1	Immediate-early transcriptional regulator UL123	DNA/RNA processing
			NP_612747.1	Glycoprotein UL124	Poorly characterized
			NP_612754.1	Putative CXC chemokine UL147	Immune system modulation
			NP_612755.1	Putative CXC chemokine UL146A	Immune system modulation
			NP_612756.1	Putative CXC chemokine UL146	Immune system modulation
			NP_612758.1	Glycoprotein UL144 similar to TNFR	Immune system modulation
			NP_612759.1	MHC class I	Immune system modulation
			NP_612762.1	Glycoprotein UL139	Poorly characterized
			NP_612775.1	Transcriptional transactivator IRS1	DNA/RNA processing
			NP_612780.1	Glycoprotein US7	Poorly characterized
			NP_612782.1	Glycoprotein US9	Poorly characterized
			NP_612783.1	Glycoprotein US10	Poorly characterized
			NP_612784.1	Glycoprotein US11 targetting MHC class I molecules for proteasome-dependent degradation; US6 family	Immune system modulation
			NP_612799.1	Chemokine receptor homologue	Immune system modulation
			NP_612807.1	Transcriptional transactivator TRS1	DNA/RNA processing
				Genes of unknown function: 26	
	Human herpesvirus 6B	NC_000898.1	NP_597817.1	G-protein coupled receptor homolog	Immune system modulation
			NP_050262.1	Chemokine	Immune system modulation
			NP_050264.1	OX-2 homolog	Immune system modulation
				Genes of unknown function: 3	

Classification	Species	Genome ID	Protein ID	Annotation	Functional Category
	Murid herpesvirus 1	NC_004065.1	YP_214012.1	Glycoprotein family m02	Poorly characterized
			YP_214013.1	Glycoprotein family m02	Poorly characterized
			YP_214016.1	Glycoprotein family m02	Poorly characterized
			YP_214017.1	Glycoprotein family m02	Poorly characterized
			YP_214018.1	Glycoprotein family m02	Poorly characterized
			YP_214019.1	Glycoprotein family m02	Poorly characterized
			YP_214020.1	Glycoprotein family m02	Poorly characterized
			YP_214021.1	Glycoprotein family m02	Poorly characterized
			YP_214022.1	Glycoprotein family m02	Poorly characterized
			YP_214023.1	Glycoprotein family m02	Poorly characterized
			YP_214024.1	Glycoprotein family m02	Poorly characterized
			YP_214111.1	Serine-alanine-rich glycoprotein	Poorly characterized
				Genes of unknown function: 40	
	Human herpesvirus 5 strain AD169	NC_001347.2	NP_899637.1	IgG Fc-binding glycoprotein RL11	Immune system modulation
			NP_899638.1	IgG Fc-binding glycoprotein RL12	Immune system modulation
			NP_039943.1	Membrane protein precursor	Poorly characterized
			NP_039945.1	Membrane protein precursor	Poorly characterized
			NP_039952.1	MHC class I antigen	Immune system modulation
			NP_783776.1	Secreted glycoprotein	Poorly characterized
			NP_039971.1	Viral mitochondrial inhibitor of apoptosis (vMIA)	Immune system modulation
			NP_039984.1	Membrane-associated protein involved in egress of capsids from nucleus	Entry and exit
			NP_040007.1	Envelope glycoprotein UL73	Viral particle associated
			NP_040008.1	Glycoprotein UL7	Poorly characterized
			NP_040050.1	Early phosphoprotein	Poorly characterized
			NP_040058.1	Membrane protein UL121	Poorly characterized
			NP_040060.1	Major immediate-early protein	Poorly characterized
			NP_040061.1	Glycoprotein UL124	Poorly characterized
			NP_040067.1	Putative secreted protein	Poorly characterized
			NP_899641.1	Glycoprotein RL1	Poorly characterized
			NP_899642.1	IgG Fc-binding glycoprotein RL11	Immune system modulation
			NP_040085.1	Transcriptional transactivator IRS1	DNA/RNA processing
			NP_040088.1	Immediate-early glycoprotein US3	Poorly characterized
			NP_040091.1	Glycoprotein US6	Poorly characterized
			NP_040092.1	Glycoprotein US7	Poorly characterized
			NP_040094.1	Glycoprotein US9	Poorly characterized
			NP_040095.1	Glycoprotein US10	Poorly characterized
			NP_040096.1	Glycoprotein US11	Poorly characterized
				Genes of unknown function: 29	
	Human herpesvirus 6	NC_001664.1		Genes of unknown function: 11	
	Human herpesvirus 7	NC_001716.2	YP_073787.1	Envelope lycoprotein U47	Viral particle associated
			YP_073812.1	Envelope lycoprotein M	Viral particle associated
				Genes of unknown function: 6	
	Cercopithecine herpesvirus 8	NC_006150.1	YP_068195.1	Envelope glycoprotein	Viral particle associated
				Genes of unknown function: 79	

Classification	Species	Genome ID	Protein ID	Annotation	Functional Category
	Murid herpesvirus 2	NC_002512.2	NP_064173.1	Putative membrane protein	Poorly characterized
			NP_064217.1	Glycoprotein L precursor	Poorly characterized
			NP_064218.1	Mucin (Fragment)	Poorly characterized
			NP_064229.1	Large tegument protein	Viral particle associated
			NP_064238.1	Nonstructural protein NS	Poorly characterized
			NP_064239.1	Immediate-early protein 2 (IE2)	Poorly characterized
			NP_064240.1	CC chemokine homolog	Immune system modulation
			NP_064247.1	Tegument protein	Viral particle associated
			NP_064248.1	Tegument protein	Viral particle associated
			NP_064249.1	Tegument protein	Viral particle associated
			NP_064251.1	MHC class Ib antigen	Immune system modulation
				Genes of unknown function: 76	
	Human herpesvirus 5 strain Merlin	NC_006273.1	YP_081459.1	IgG Fc-binding glycoprotein RL11	Immune system modulation
			YP_081460.1	IgG Fc-binding glycoprotein RL12	Immune system modulation
			YP_081461.1	Glycoprotein RL11 family member	Poorly characterized
			YP_081462.1	Glycoprotein RL11 family member	Poorly characterized
			YP_081466.1	Glycoprotein RL11 family member	Poorly characterized
			YP_081467.1	Glycoprotein RL11 family member	Poorly characterized
			YP_081468.1	Glycoprotein RL11 family member	Poorly characterized
			YP_081469.1	Glycoprotein RL11 family member	Poorly characterized
			YP_081470.1	Glycoprotein RL11 family member	Poorly characterized
			YP_081471.1	Glycoprotein RL11 family member	Poorly characterized
			YP_081477.1	MHC class I-like protein UL18	Immune system modulation
			YP_081479.1	Glycoprotein RL11 family member	Poorly characterized
			YP_081481.1	Glycoprotein	Poorly characterized
			YP_081496.1	Viral mitochondrial inhibitor of apoptosis (vMIA)	Immune system modulation
			YP_081509.1	Membrane-associated protein involved in egress of capsids from nucleus	Entry and exit
			YP_081521.1	Envelope glycoprotein UL73	Viral particle associated
			YP_081522.1	Glycoprotein O	Poorly characterized
			YP_081529.1	Protease and minor capsid scaffold protein UL80	Viral particle formation
			YP_081542.1	Tegument protein UL95	Viral particle associated
			YP_081553.1	UL112 involved in recruiting DNA replication proteins to nuclear replication compartments	DNA/RNA processing
			YP_081559.1	Glycoprotein	Poorly characterized
			YP_081560.1	Membrane protein	Poorly characterized
			YP_081562.1	Immediate-early transcriptional regulator UL123	DNA/RNA processing
			YP_081563.1	Glycoprotein	Poorly characterized
			YP_081565.1	Putative secreted protein	Poorly characterized
			YP_081570.1	Putative CXC chemokine UL147	Immune system modulation
			YP_081571.1	Alpha-chemokine; UL146 family member	Immune system modulation
			YP_081573.1	Glycoprotein similar to TNFR	Immune system modulation
			YP_081574.1	Similar to MHC class I; UL18 family member	Immune system modulation
			YP_081577.1	Glycoprotein	Poorly characterized
			YP_081587.1	Transcriptional transactivator IRS1	DNA/RNA processing
			YP_081590.1	Immediate-early glycoprotein US3	Poorly characterized
			YP_081591.1	Glycoprotein; US6 family member	Poorly characterized
			YP_081592.1	Glycoprotein; US6 family member	Poorly characterized
			YP_081594.1	Glycoprotein; role in cell-to-cell spread in epithelial cells; US6 family member	Poorly characterized
			YP_081595.1	Glycoprotein; US6 family member	Poorly characterized
				Genes of unknown function: 20	

Classification	Species	Genome ID	Protein ID	Annotation	Functional Category
	Tupaiid herpesvirus 1	NC_002794.1	NP_116415.1	Genes of unknown function: 59	
Gammaherpesvirus					
	Human herpesvirus 4	NC_007605.1	YP_401631.1	Latent membrane protein 2 (Terminal protein)	Immune system modulation
			YP_401632.1	Latent membrane protein 2 (Terminal protein)	Immune system modulation
			YP_401634.1	Viral interleukin-10 homolog precursor (vIL-10)	Immune system modulation
			YP_401644.1	Epstein-Barr nuclear antigen 2 (EBV nuclear antigen 2) (EBNA-2)	Poorly characterized
			YP_401646.1	Early antigen protein R (EA-R) (Nuclear antigen)Bcl-2 homolog (BHRF1)	Immune system modulation
			YP_401667.1	BLLF1 envelope glycoprotein	Entry and exit
			YP_401669.1	Epstein-Barr nuclear antigen 3 (EBNA-3A), function in nucleotide salvage pathway	Nucleotide metabolism
			YP_401670.1	Epstein-Barr nuclear antigen (EBNA-3B)	DNA/RNA processing
			YP_401671.1	Epstein-Barr nuclear antigen (EBNA-3C)	DNA/RNA processing
			YP_401673.1	Trans-activator protein BZLF1 (EB; Zebra)	DNA/RNA processing
			YP_401677.1	Epstein-Barr nuclear antigen 1 (EBNA-1)	DNA/RNA processing
			YP_401706.1	Membrane glycoprotein	Poorly characterized
			YP_401709.1	dUTPase	Nucleotide metabolism
			YP_401722.1	Latent membrane protein 1 (LMP-1) (Protein p63)	Immune system modulation
				Genes of unknown function: 23	
	Equid herpesvirus 2	NC_001650.1	NP_042597.1	C-C chemokine receptor type 3	Immune system modulation
			NP_042604.1	Glycoprotein B	Poorly characterized
			NP_042618.1	Glycoprotein H	Poorly characterized
			NP_042628.1	Viral DNA cleavage/packaging protein	Viral particle formation
			NP_042633.1	Probable serine/threonine-protein kinase	Poorly characterized
			NP_042643.1	Uracil-DNA glycosylase	DNA/RNA processing
			NP_042644.1	Glycoprotein L precursor	Poorly characterized
			NP_042645.1	Glycoprotein L	Poorly characterized
			NP_042654.1	Post-transcriptional transactivator	DNA/RNA processing
			NP_042661.1	Tegument protein	Viral particle associated
			NP_042662.1	Capsid protein	Viral particle associated
			NP_042670.1	G protein-coupled receptor	Immune system modulation
			NP_042674.1	Probable C-C chemokine receptor type 3	Immune system modulation
			NP_042675.1	Probable C-C chemokine receptor type 3	Immune system modulation
				Genes of unknown function: 14	
	Cercopithecine herpesvirus 15	NC_006146.1	YP_067938.1	Terminal protein LMP2A	Immune system modulation
			YP_067939.1	Terminal protein LMP2A	Immune system modulation
			YP_067944.1	Bcl-2 homolog (BHRF1)	Immune system modulation
			YP_067963.1	gp350 envelope glycoprotein	Entry and exit
			YP_067965.1	Epstein-Barr nuclear antigen 3 (EBNA-3A), function in nucleotide salvage pathway	Nucleotide metabolism
			YP_067966.1	Nuclear antigen 3B (EBNA-3B)	DNA/RNA processing
			YP_067967.1	Nuclear antigen 3C (EBNA-3C)	DNA/RNA processing
			YP_067969.1	Trans-activator protein BZLF1	DNA/RNA processing
			YP_067973.1	Nuclear antigen EBNA-1	DNA/RNA processing
			YP_067991.1	Putative membrane antigen gp85	Poorly characterized
			YP_068002.1	Probable membrane glycoprotei	Poorly characterized
			YP_068017.1	Latent membrane protein 1 (LMP-1)	Immune system modulation
				Genes of unknown function: 10	

Classification	Species	Genome ID	Protein ID	Annotation	Functional Category
Unclassified herpesvirus					
	Ostreid herpesvirus 1	NC_005881.1	YP_024582.1	RING finger protein	Poorly characterized
			YP_024600.1	Hypothetical protein (Mid-1-related chloride channel 1)	Poorly characterized
			YP_024619.1	Membrane protein	Poorly characterized
				Genes of unknown function: 4	
Caudovirales					
	Enterobacteria phage RB43	NC_007023.1	YP_239022.1	gp59, loader of gene 41 DNA helicase	DNA/RNA processing
			YP_239162.1	MutT (NUDIX) domain	DNA/RNA processing
			YP_239174.1	dNMP kinase	Nucleotide metabolism
			YP_239192.1	MobE mobile endonuclease	Mobile elements
			YP_239258.1	gp38 distal long tail fiber assembly catalyst	Viral particle formation
			YP_239259.1	t holin	Entry and exit
				Genes of unknown function: 9	
	Enterobacteria phage T4	NC_000866.4	NP_049856.1	gp59, loader of gene 41 DNA helicase	DNA/RNA processing
			NP_049861.1	gp35 hinge connector of long tail fiber, proximal connector	Viral particle associated
			NP_049865.1	t holin lysis mediator	Entry and exit
			NP_049887.1	DenB DNA endonuclease	DNA/RNA processing
				Genes of unknown function: 4	
	Pseudomonas phage phiEL	NC_007623.1	YP_418058.1	HNH endonuclease	Mobile elements
			YP_418070.1	Putative transposase	Mobile elements
			YP_418185.1	Putative acetyltransferase	Other metabolism
				Genes of unknown function: 15	
	Pseudomonas phage phiKZ	NC_004629.1	NP_803862.1	HNH endonuclease	Mobile elements
				Genes of unknown function: 26	
	Mycobacterium phage Bxz1	NC_004687.1	NP_818246.1	Putative DNA primase	DNA/RNA processing
				Genes of unknown function: 26	
	Enterobacteria phage RB69	NC_004928.1	NP_861748.1	gp62 clamp loader subunit, DNA polymerase accessory protein	DNA/RNA processing
			NP_861804.1	Valyl-tRNA synthetase modifier	Translation and protein metabolism
			NP_861937.1	gp59, loader of gp41 DNA helicase	DNA/RNA processing
			NP_861942.1	gp35 hinge connector of long tail fiber, proximal connector	Viral particle associated
			NP_861946.1	t holin lysis mediator	Entry and exit
				Genes of unknown function: 6	
	Enterobacteria phage RB49	NC_005066.1		Genes of unknown function: 12	
	Vibrio phage KVP40	NC_005083.1	NP_899252.1	gp59, loader of gp41 DNA helicase	DNA/RNA processing
			NP_899254.1	Dihydrofolate reductase	Nucleotide metabolism
				Genes of unknown function: 16	
	Aeromonas phage 44RR2.8t	NC_005135.1	NP_932385.1	DNA replication primase	DNA/RNA processing
			NP_932560.1	Endonuclease II	DNA/RNA processing
			NP_932562.1	Dihydrofolate reductase	Nucleotide metabolism
				Genes of unknown function: 4	
	Aeromonas phage Aeh1	NC_005260.1	NP_943926.1	MobE homing endonuclease	Mobile elements
			NP_944074.1	dNMP kinase	Nucleotide metabolism
			NP_944082.1	gp53 baseplate wedge subunit	Viral particle associated
				Genes of unknown function: 10	

Classification	Species	Genome ID	Protein ID	Annotation	Functional Category
	Bacteriophage S-PM2	NC_006820.1	YP_195035.1	Site-specific DNA methylase Dam	DNA/RNA processing
			YP_195130.1	Terminase subunit gp16	Viral particle formation
			YP_195144.1	Tail completion protein gp3	Viral particle formation
			YP_195148.1	Sigma factor for late transcription gp5	DNA/RNA processing
			YP_195157.1	Sliding clamp loader gp44	DNA/RNA processing
			YP_195161.1	Putative cytosine-specific methyl transferase, C-terminal portion	DNA/RNA processing
			YP_195165.1	Small heat shock protein	Translation and protein metabolism
			YP_195211.1	Photosystem II D1 protein	Other metabolism
			YP_195214.1	Photosystem II D2 protein	Other metabolism
			YP_195238.1	Head completion gp4	Viral particle formation
			YP_195267.1	Putative helicase	DNA/RNA processing
				Genes of unknown function: 17	
	Cyanophage P-SSM2	NC_006883.1	YP_214238.1	T4-like loader of gp41 DNA helicase	DNA/RNA processing
			YP_214271.1	Helicase	DNA/RNA processing
			YP_214340.1	Dioxygenase	Other metabolism
			YP_214341.1	T4-like baseplate wedge	Viral particle associated
			YP_214374.1	RNA-DNA / DNA-DNA helicase ATPase	DNA/RNA processing
			YP_214393.1	T4-like clamp loader subunit	DNA/RNA processing
			YP_214417.1	Recombination and repair protein	DNA/RNA processing
			YP_214418.1	T4-like recA-like recombination protein	DNA/RNA processing
			YP_214438.1	T4-like DNA primase	DNA/RNA processing
			YP_214439.1	Ribonucleoside-diphosphate reductase	Nucleotide metabolism
			YP_214490.1	NDP sugar epimerase/dehydratase	Other metabolism
				Genes of unknown function: 19	
	Cyanophage P-SSM4	NC_006884.1	YP_214609.1	N4C methyltransferase possible T4-like gp42 homologue	DNA/RNA processing
			YP_214637.1	Redox protein	Other metabolism
			YP_214638.1	T4-like pyrimidine DNA glycosylase DenV	DNA/RNA processing
			YP_214650.1	gp14 T4-like neck protein	Viral particle associated
			YP_214658.1	Putative oxidoreductase	Other metabolism
			YP_214677.1	RNA-DNA / DNA-DNA helicase ATPase, UvsW	DNA/RNA processing
			YP_214679.1	gp55 T4-like sigma factor late transxn	DNA/RNA processing
			YP_214680.1	gp47 T4-like endonuclease	DNA/RNA processing
			YP_214682.1	gp46 T4-like endonuclease	DNA/RNA processing
			YP_214684.1	PcyA, phycocyanobilin:ferredoxin oxidoreductase	Other metabolism
			YP_214691.1	gp62 T4-like clamp loader subunit	DNA/RNA processing
			YP_214692.1	Translation repressor protein	Translation and protein metabolism
			YP_214708.1	T4-like recA-like recombination protein, UvsX	DNA/RNA processing
			YP_214726.1	gp61 T4-like DNA primase	DNA/RNA processing
			YP_214732.1	TalC, transaldolase	Other metabolism
				Genes of unknown function: 5	
	Aeromonas phage 31	NC_007022.1	YP_238758.1	gp61 T4-like DNA primase	DNA/RNA processing
			YP_238890.1	Putative packaged DNA stabilization protein	Viral particle associated
			YP_238934.1	Dihydrofolate reductase	Nucleotide metabolism
				Genes of unknown function: 7	
	Bacteriophage c-st	NC_007581.1	YP_398521.1	Putative uracil permease	Nucleotide metabolism
			YP_398612.1	Putative IS transposase (OrfA)	Mobile elements
				Genes of unknown function: 6	
