# Supplementary material for: Horizontal gene transfer and nucleotide compositional anomaly in large DNA viruses
Source: BMC Genomics. 2007 Dec 10;8:456. doi: 10.1186/1471-2164-8-456 (PMC2211322; doi:10.1186/1471-2164-8-456)
Supplement: Additional file 9 — Summary of viral genes involved in the control of host defense system. [file 1471-2164-8-456-S9.pdf]

Summary of viral genes involved in the control of host defense system.

	Innate Immune System			Adaptive Immune System			
	**Cytokine**	**Chemokine**	**MHC Class I Homologs**	**Antigen Presentation Pathway**	**Antibody Interference**	**Apoptosis**	**Other Pathways**
**Poxvirus**	IL-10; IL-18-BP; IL-1 β-R; INF-R; TNF-R; CD30; CD150 (SLAM); CD200 (OX-2)	Chemokine; Chemokine-R	MHC class I	LAP domain (MHC class I down regulation)		Ankyrin; Bcl-2; FLIP; dsRNA-biding protein; eIF2α; M-T4; Apoptosis regulator M11L; Glutathione peroxidase; Serpin; C-type lectin	Semaphorines; Complement-binding protein; CD47
**α-herpesvirus**				ICP47 (inhibits peptide loading)	Fc-binding protein	ICP34.5 (GADD34); Glycoprotein J; RNA-binding protein interacting with PKR	Complement-regulatory protein
**β-herpesvirus**	IL-10; TNF-R; CD200 (OX-2)	Chemokine; Chemokine-R	MHC class I	MCMV/gp48 (directs MHC class I to lysosome); MCMV/gp40 (downregulates MHC class I); HCMV/US11 (causes degradation of MHC class I)	Fc-binding protein	vMIA	
**γ-herpesvirus**	IL-10; IL-6; INF regulatory factor (ICSBP)	Chemokine; Chemokine-R		KSHV/K3,K5 (induce endocytosis of MHC class I)		Bcl-2; FLIP	CCP (complement control protein); LMP-1; LMP-2A
**Asfarvirus**						C-type lectin; IAP; Bcl-2	IκB (Inhibotor of NFκB)
**Baculovirus**						Inhibitor of apoptosis	
**Iridovirus**						Caspase-1	
**Mimivirus**						**EI24**	

For those highlighted by red, our analyses identified several genes in the 67 LDV genomes as cA genes. For EI24 (in blue), see text.
