# Supplementary material for: Horizontal gene transfer and nucleotide compositional anomaly in large DNA viruses
Source: BMC Genomics. 2007 Dec 10;8:456. doi: 10.1186/1471-2164-8-456 (PMC2211322; doi:10.1186/1471-2164-8-456)
Supplement: Additional file 10 — Phylogenetic trees supporting the HGT events for the cA genes detected by a composition-based approach. (A) Glutathione peroxidase, (B) Interleukin 10, (C) R1R hypothetical protein, (D) G protein-coupled receptor, (E) Photosystem II D2 protein, (F) Site-specific DNA methylase, (G) Thymidylate synthase and (H) Cytosine methyltransferase. Each node is labeled with SWISS-PROT/TrEMBL entry, the taxonomic group (A for Archaea, B for Bacteria, E for Eukaryota and V for Virus) and the specie name. The viral genes detected as cA genes by the method of the Nakamura et al. are highlighted in red. "I" corresponds to a clade branching with the viral cA genes; "O1" corresponds to the outgroup of I, and "O2" corresponds to the outgroup of I and O1. Paralogs, orthologs and homologs indicate the relationships between the outgroups (O1 or O2) and the internal groups (I or (I, O1)). (A-E) satisfy the criterion 1, and (F-H) satisfy the criterion 2 (see Methods). Tree reconstruction was carried out by the maximum likelihood method. Bootstrap values are indicated as the branch levels. [file 1471-2164-8-456-S10.pdf]

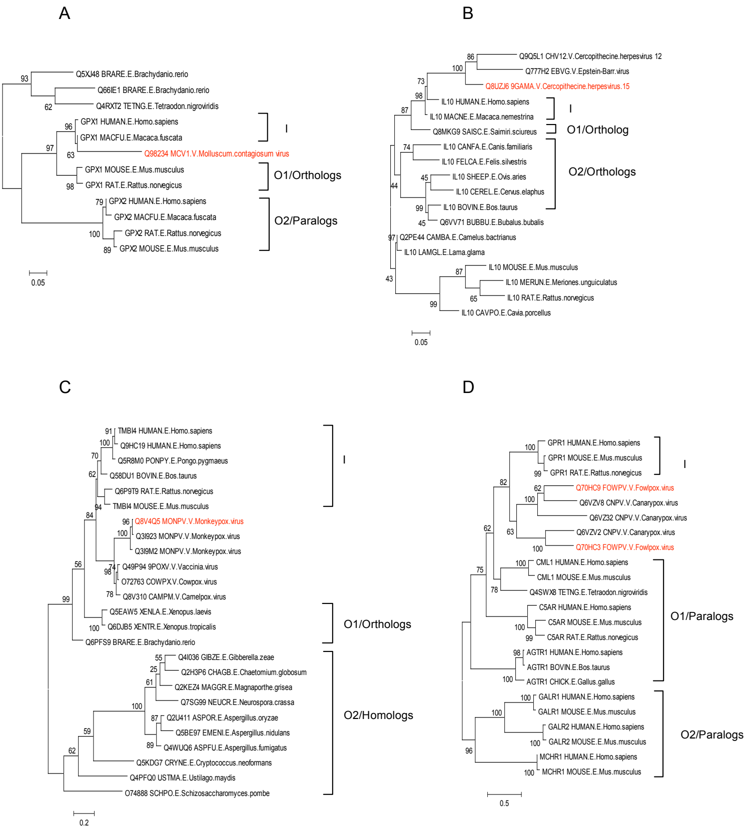
A
Q5XJ48 BRARE.E.Brachydanio.rerio
Q66IE1 BRARE.E.Brachydanio.rerio
Q4RXT2 TETNG.E.Tetraodon.nigroviridis
GPX1 HUMAN.E.Homo.sapiens
GPX1 MACFU.E.Macaca.fuscata
Q98234 MCV1.V.Molluscum.contagiosum virus
GPX1 MOUSE.E.Mus.musculus
GPX1 RAT.E.Rattus.norvegicus
GPX2 HUMAN.E.Homo.sapiens
GPX2 MACFU.E.Macaca.fuscata
GPX2 RAT.E.Rattus.norvegicus
GPX2 MOUSE.E.Mus.musculus
I
O1/Orthologs
O2/Paralogs
0.05
B
Q9Q5L1 CHV12.V.Cercopithecine.herpesvirus 12
Q777H2 EBVG.V.Epstein-Barr.virus
Q8UZJ6 9GAMA.V.Cercopithecine.herpesvirus.15
IL10 HUMAN.E.Homo.sapiens
IL10 MACNE.E.Macaca.nemestrina
Q8MKG9 SAISC.E.Saimiri.sciureus
IL10 CANFA.E.Canis.familiaris
IL10 FELCA.E.Felis.silvestris
IL10 SHEEP.E.Ovis.aries
IL10 CEREL.E.Cervus.elaphus
IL10 BOVIN.E.Bos.taurus
Q6VV71 BUBBU.E.Bubalus.bubalis
Q2PE44 CAMBA.E.Camelus.bactrianus
IL10 LAMGL.E.Lama.glama
IL10 MOUSE.E.Mus.musculus
IL10 MERUN.E.Meriones.unguiculatus
IL10 RAT.E.Rattus.norvegicus
IL10 CAVPO.E.Cavia.porcellus
I
O1/Ortholog
O2/Orthologs
0.05
C
TMBI4 HUMAN.E.Homo.sapiens
Q9HC19 HUMAN.E.Homo.sapiens
Q5R8M0 PONPY.E.Pongo.pygmaeus
Q58DU1 BOVIN.E.Bos.taurus
Q6P9T9 RAT.E.Rattus.norvegicus
TMBI4 MOUSE.E.Mus.musculus
Q8V4Q5 MONPV.V.Monkeypox.virus
Q3I923 MONPV.V.Monkeypox.virus
Q3I9M2 MONPV.V.Monkeypox.virus
Q49P94 9POXV.V.Vaccinia.virus
O72763 COWPX.V.Cowpox.virus
Q8V310 CAMPM.V.Camelpox.virus
Q5EAW5 XENLA.E.Xenopus.laevis
Q6DJB5 XENTR.E.Xenopus.tropicalis
Q6PFS9 BRARE.E.Brachydanio.rerio
Q4I036 GIBZE.E.Gibberella.zeae
Q2H3P6 CHAGB.E.Chaetomium.globosum
Q2KEZ4 MAGGR.E.Magnaporthe.grisea
Q7SG99 NEUCR.E.Neurospora.crassa
Q2U411 ASPOR.E.Aspergillus.oryzae
Q5BE97 EMENI.E.Aspergillus.nidulans
Q4WUQ6 ASPFU.E.Aspergillus.fumigatus
Q5KDG7 CRYNE.E.Cryptococcus.neoformans
Q4PFQ0 USTMA.E.Ustilago.maydis
O74888 SCHPO.E.Schizosaccharomyces.pombe
I
O1/Orthologs
O2/Homologs
0.2
D
GPR1 HUMAN.E.Homo.sapiens
GPR1 MOUSE.E.Mus.musculus
GPR1 RAT.E.Rattus.norvegicus
Q70HC9 FOWPV.V.Fowlpox.virus
Q6VZV8 CNPV.V.Canarypox.virus
Q6VZ32 CNPV.V.Canarypox.virus
Q6VZV2 CNPV.V.Canarypox.virus
Q70HC3 FOWPV.V.Fowlpox.virus
CML1 HUMAN.E.Homo.sapiens
CML1 MOUSE.E.Mus.musculus
Q4SWX8 TETNG.E.Tetraodon.nigroviridis
C5AR HUMAN.E.Homo.sapiens
C5AR MOUSE.E.Mus.musculus
C5AR RAT.E.Rattus.norvegicus
AGTR1 HUMAN.E.Homo.sapiens
AGTR1 BOVIN.E.Bos.taurus
AGTR1 CHICK.E.Gallus.gallus
GALR1 HUMAN.E.Homo.sapiens
GALR1 MOUSE.E.Mus.musculus
GALR2 HUMAN.E.Homo.sapiens
GALR2 MOUSE.E.Mus.musculus
MCHR1 HUMAN.E.Homo.sapiens
MCHR1 MOUSE.E.Mus.musculus
I
O1/Paralogs
O2/Paralogs
0.5

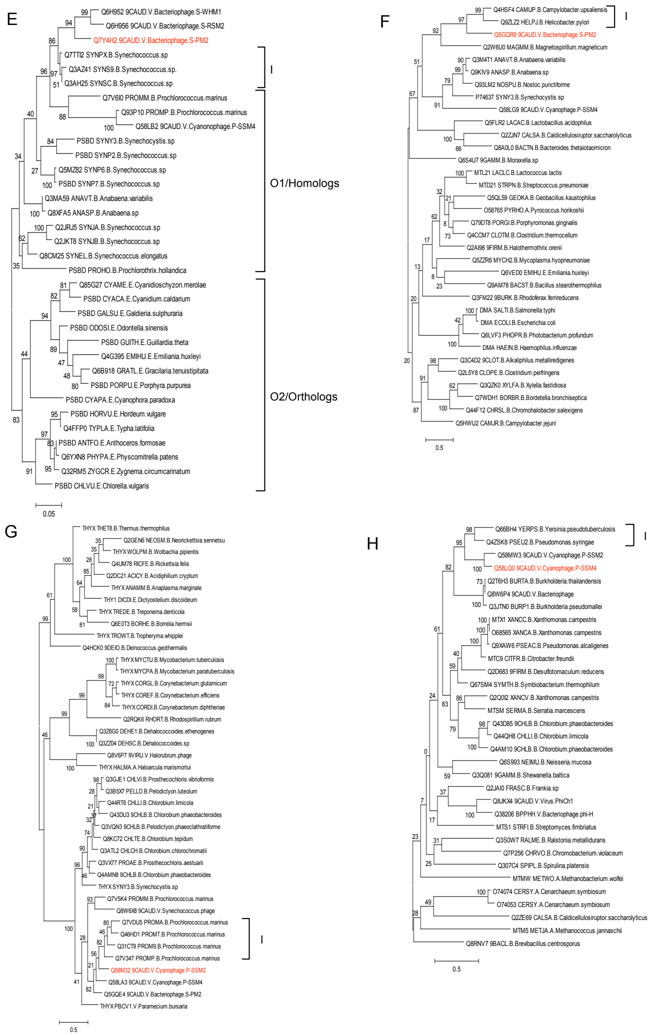
E
Q6H952 9CAUD.V.Bacteriophage.S-WHM1
Q6H956 9CAUD.V.Bacteriophage.S-RSM2
Q7Y4H2 9CAUD.V.Bacteriophage.S-PM2
Q7TTI2 SYNPX.B.Synechococcus.sp
Q3AZ41 SYNS9.B.Synechococcus.sp.
Q3AH25 SYNSC.B.Synechococcus.sp
I
Q7V6I0 PROMM.B.Prochlorococcus.marinus
Q93P10 PROMP.B.Prochlorococcus.marinus
Q58LB2 9CAUD.V.Cyanonophage.P-SSM4
PSBD SYNY3.B.Synechocystis.sp
PSBD SYNP2.B.Synechococcus.sp
Q5MZ82 SYNP6.B.Synechococcus.sp
PSBD SYNP7.B.Synechococcus.sp
O1/Homologs
Q3MA59 ANAVT.B.Anabaena.variabilis
Q8XFA5 ANASP.B.Anabaena.sp
Q2JRJ5 SYNJA.B.Synechococcus.sp
Q2JKT8 SYNJB.B.Synechococcus.sp
Q8CM25 SYNEL.B.Synechococcus.elongatus
PSBD PROHO.B.Prochlorothrix.hollandica
Q85G27 CYAME.E.Cyanidioschyzon.merolae
PSBD CYACA.E.Cyanidium.caldarium
PSBD GALSU.E.Galdieria.sulphuraria
PSBD ODOSI.E.Odontella.sinensis
PSBD GUITH.E.Guillardia.theta
Q4G395 EMIHU.E.Emiliania.huxleyi
Q6B918 GRATL.E.Gracilaria.tenuistipitata
PSBD PORPU.E.Porphyra.purpurea
O2/Orthologs
PSBD CYAPA.E.Cyanophora.paradoxa
PSBD HORVU.E.Hordeum.vulgare
Q4FFP0 TYPLA.E.Typha.latifolia
PSBD ANTFO.E.Anthoceros.formosae
Q6YXN8 PHYPA.E.Physcomitrella.patens
Q32RM5 ZYGCR.E.Zygnema.circumcarinatum
PSBD CHLVU.E.Chlorella.vulgaris
0.05
F
Q4HSF4 CAMUP.B.Campylobacter.upsaliensis
Q9ZLZ2 HELPJ.B.Helicobacter.pylori
I
Q5GQR9 9CAUD.V.Bacteriophage.S-PM2
Q2W6U0 MAGMM.B.Magnetospirillum.magneticum
Q3M4T1 ANAVT.B.Anabaena.variabilis
Q9KIV9 ANASP.B.Anabaena.sp
Q93LM2 NOSPU.B.Nostoc.punctiforme
P74637 SYNY3.B.Synechocystis.sp
Q58LG9 9CAUD.V.Cyanophage.P-SSM4
Q5FLR2 LACAC.B.Lactobacillus.acidophilus
Q2ZJN7 CALSA.B.Caldicellulosiruptor.saccharolyticus
Q8A0L0 BACTN.B.Bacteroides.thetaiotaomicron
Q6S4U7 9GAMM.B.Moraxella.sp
MTL21 LACLC.B.Lactococcus.lactis
MTD21 STRPN.B.Streptococcus.pneumoniae
Q5QL59 GEOKA.B.Geobacillus.kaustophilus
O58765 PYRHO.A.Pyrococcus.horikoshii
Q79DT8 PORGI.B.Porphyromonas.gingivalis
Q4CCM7 CLOTM.B.Clostridium.thermocellum
Q2AI98 9FIRM.B.Halothermothrix.orenii
Q5ZZR6 MYCH2.B.Mycoplasma.hyopneumoniae
Q6VED0 EMIHU.E.Emiliania.huxleyi
Q9AM78 BACST.B.Bacillus.stearothermophilus
Q3FM22 9BURK.B.Rhodoferax.ferrireducens
DMA SALTI.B.Salmonella.typhi
DMA ECOLI.B.Escherichia.coli
Q6LVF3 PHOPR.B.Photobacterium.profundum
DMA HAEIN.B.Haemophilus.influenzae
Q3C4D2 9CLOT.B.Alkaliphilus.metalliredigenes
Q2L5Y8 CLOPE.B.Clostridium.perfringens
Q3QZK0 XYLFA.B.Xylella.fastidiosa
Q7WDH1 BORBR.B.Bordetella.bronchiseptica
Q44F12 CHRSL.B.Chromohalobacter.salexigens
Q5HWU2 CAMJR.B.Campylobacter.jejuni
0.5
G
THYX THET8.B.Thermus.thermophilus
Q2GEN6 NEOSM.B.Neorickettsia.sennetsu
THYX WOLPM.B.Wolbachia.pipientis
Q4UM78 RICFE.B.Rickettsia.felis
Q2DC21 ACICY.B.Acidiphilium.cryptum
THYX ANAMM.B.Anaplasma.marginale
THY1 DICDI.E.Dictyostelium.discoideum
THYX TREDE.B.Treponema.denticola
Q6E0T3 BORHE.B.Borrelia.hermsii
THYX TROWT.B.Tropheryma.whipplei
Q4HCK0 9DEIO.B.Deinococcus.geothermalis
THYX MYCTU.B.Mycobacterium.tuberculosis
THYX MYCPA.B.Mycobacterium.paratuberculosis
THYX CORGL.B.Corynebacterium.glutamicum
THYX COREF.B.Corynebacterium.efficiens
THYX CORDI.B.Corynebacterium.diphtheriae
Q2RQK6 RHORT.B.Rhodospirillum.rubrum
Q3Z6G0 DEHE1.B.Dehalococcoides.ethenogenes
Q3ZZ04 DEHSC.B.Dehalococcoides.sp
Q8V6P7 9VIRU.V.Halorubrum.phage
THYX HALMA.A.Haloarcula.marismortui
Q3GJE1 CHLVI.B.Prosthecochloris.vibrioformis
Q3B5X7 PELLD.B.Pelodictyon.luteolum
Q44RT6 CHLLI.B.Chlorobium.limicola
Q43DU3 9CHLB.B.Chlorobium.phaeobacteroides
Q3VQN3 9CHLB.B.Pelodictyon.phaeoclathratiforme
Q8KC72 CHLTE.B.Chlorobium.tepidum
Q3ATL2 CHLCH.B.Chlorobium.chlorochromatii
Q3VX77 PROAE.B.Prosthecochloris.aestuarii
Q4AMN8 9CHLB.B.Chlorobium.phaeobacteroides
THYX SYNY3.B.Synechocystis.sp
Q7V5K4 PROMM.B.Prochlorococcus.marinus
Q8W6X8 9CAUD.V.Synechococcus.phage
Q7VDU5 PROMA.B.Prochlorococcus.marinus
Q46HD1 PROMT.B.Prochlorococcus.marinus
Q31CT9 PROM9.B.Prochlorococcus.marinus
Q7V347 PROMP.B.Prochlorococcus.marinus
I
Q58M32 9CAUD.V.Cyanophage.P-SSM2
Q58LA3 9CAUD.V.Cyanophage.P-SSM4
Q5GQE4 9CAUD.V.Bacteriophage.S-PM2
THYX PBCV1.V.Paramecium.bursaria
0.5
H
Q66BH4 YERPS.B.Yersinia.pseudotuberculosis
Q4ZSK8 PSEU2.B.Pseudomonas.syringae
I
Q58MW3 9CAUD.V.Cyanophage.P-SSM2
Q58LQ0 9CAUD.V.Cyanophage.P-SSM4
Q2T6H3 BURTA.B.Burkholderia.thailandensis
Q8W6P4 9CAUD.V.Bacteriophage
Q3JTN0 BURP1.B.Burkholderia.pseudomallei
MTX1 XANCC.B.Xanthomonas.campestris
O68565 XANCA.B.Xanthomonas.campestris
Q9XAW6 PSEAC.B.Pseudomonas.alcaligenes
MTC9 CITFR.B.Citrobacter.freundii
Q2D683 9FIRM.B.Desulfotomaculum.reducens
Q67SM4 SYMTH.B.Symbiobacterium.thermophilum
Q2Q0I2 XANCV.B.Xanthomonas.campestris
MTSM SERMA.B.Serratia.marcescens
Q43D85 9CHLB.B.Chlorobium.phaeobacteroides
Q44QH8 CHLLI.B.Chlorobium.limicola
Q4AM10 9CHLB.B.Chlorobium.phaeobacteroides
Q6S993 NEIMU.B.Neisseria.mucosa
Q3Q081 9GAMM.B.Shewanella.baltica
Q2JAI0 FRASC.B.Frankia.sp
Q8JKX4 9CAUD.V.Virus.PhiCh1
Q38206 BPPHH.V.Bacteriophage.phi-H
MTS1 STRFI.B.Streptomyces.fimbriatus
Q3S0W7 RALME.B.Ralstonia.metallidurans
Q7P256 CHRVO.B.Chromobacterium.violaceum
Q307C4 SPIPL.B.Spirulina.platensis
MTMW METWO.A.Methanobacterium.wolfei
O74074 CERSY.A.Cenarchaeum.symbiosum
O74053 CERSY.A.Cenarchaeum.symbiosum
Q2ZE69 CALSA.B.Caldicellulosiruptor.saccharolyticus
MTM5 METJA.A.Methanococcus.jannaschii
Q8RNV7 9BACL.B.Brevibacillus.centrosporus
0.5
