# Supplementary material for: Horizontal gene transfer and nucleotide compositional anomaly in large DNA viruses
Source: BMC Genomics. 2007 Dec 10;8:456. doi: 10.1186/1471-2164-8-456 (PMC2211322; doi:10.1186/1471-2164-8-456)
Supplement: Additional file 11 — Nucleotide sequence data for the hosts (or its close relatives) of the 67 LDVs. [file 1471-2164-8-456-S11.pdf]

Nucleotide sequence data for the hosts (or its close relatives) of the 67 LDVs.

Virus	Representative Host	Organisms Group Related to Host	Number of Non-redundant CDS	Total Number of Nucleotides
African swine fever virus	*Sus Scrofa*		887	1169069
Mamestra configurata NPV-A	*Mamestra configurata*	*Lepidoptera*	899	1135214
Mamestra configurata NPV-B	*Mamestra configurata*	*Lepidoptera*	899	1135214
Lymantria dispar MNPV	*Lymantria dispar*	*Lepidoptera*	899	1135214
Xestia c-nigrum granulovirus	*Xestia nigrum*	*Lepidoptera*	899	1135214
Enterobacteria phage RB43	*Enterobacteria*	*Escherichia coli*	3862	3978366
Enterobacteria phage T4	*Enterobacteria*	*Escherichia coli*	3862	3978366
Pseudomonas phage phiEL	*Pseudomonas*	-	5248	5520672
Pseudomonas phage phiKZ	*Pseudomonas*	-	5248	5520672
Mycobacterium phage Bxz1	*Mycobacterium*	-	3695	3950490
Enterobacteria phage RB69	*Enterobacteria*	*Escherichia coli*	3862	3978366
Enterobacteria phage RB49	*Enterobacteria*	*Escherichia coli*	3862	3978366
Vibrio phage KVP40	*Vibrio parahaemolyticus*	-	4161	4368585
Aeromonas phage 44RR2.8t	*Aeromonas*	-	536	537623
Aeromonas phage Aeh1	*Aeromonas*	-	536	537623
Bacteriophage S-PM2	*Synechococcus*	-	5945	6009849
Cyanophage P-SSM2	*Cyanobacteria*	*Prochlorococcus*	2779	2597712
Cyanophage P-SSM4	*Cyanobacteria*	*Prochlorococcus*	2779	2597712
Aeromonas phage 31	*Aeromonas*	-	536	537623
Bacteriophage c-st	*Clostridium botulinum*	-	22	34160
Pongine herpesvirus 4	*Simians*	*Cercopithecus*	104	139805
Human herpesvirus 4	*Homo sapiens*	-	22454	34617928
Human herpesvirus 6B	*Homo sapiens*	-	22454	34617928
Murid herpesvirus 1	*Mus musculus*	-	22626	33882315
Cercopithecine herpesvirus 16	*Simians*	*Cercopithecus*	104	139805
Human herpesvirus 5 strain AD169	*Homo sapiens*	-	22454	34617928
Equid herpesvirus 1	*Equus caballus*	-	196	244763
Equid herpesvirus 2	*Equus caballus*	-	196	244763
Cercopithecine herpesvirus 1	*Simians*	*Cercopithecus*	104	139805
Human herpesvirus 6	*Homo sapiens*	-	22454	34617928
Human herpesvirus 7	*Homo sapiens*	-	22454	34617928
Human herpesvirus 2	*Homo sapiens*	-	22454	34617928
Human herpesvirus 1	*Homo sapiens*	-	22454	34617928
Psittacid herpesvirus 1	*Psittacidae*	-	8	9649
Gallid herpesvirus 2	*Gallus gallus*	-	17526	30024643
Cercopithecine herpesvirus 15	*Simians*	*Cercopithecus*	104	139805
Cercopithecine herpesvirus 8	*Simians*	*Cercopithecus*	104	139805
Murid herpesvirus 2	*Rattus norvegicus*	-	22708	37114122
Human herpesvirus 5 strain Merlin	*Homo sapiens*	-	22454	34617928
Gallid herpesvirus 3	*Galuus gallus*	-	17526	30024643
Cercopithecine herpesvirus 2	*Simians*	*Cercopithecus*	104	139805
Meleagrid herpesvirus 1	*Meleagris gallopavo*	*Meleagris*	50	55174
Tupaiid herpesvirus 1	*Tupaia glis*	*Tupaia*	22	26589
Ostreid herpesvirus 1	*Ostreidae*	-	63	79203
Lymphocystis disease virus - isolate China	*Fish*	*Danio rerio*	6841	8893321
Invertebrate iridescent virus 6	*Invertebrate*	*Lepidoptera*	899	1135214
Acanthamoeba polyphaga mimivirus	*Acanthamoeba*	*Acanthamoeba castellanii*	24	40605
Shrimp white spot syndrome virus	*Penaeus*	-	24	38013
Emiliania huxleyi virus 86	*Emiliania huxleyi*	-	5	4824
Paramecium bursaria Chlorella virus 1	*Zoochlorella*	*Chlorella*	18	26637
Ectocarpus siliculosus virus 1	*Ectocarpus siliculosus*	*Ectocarpus*	2	2811
Monkeypox virus	*Simians / Homo sapiens*	*Homo sapiens*	22454	34617928
Camelpox virus	*Camelus*	-	25	19314
Cowpox virus	*Bos taurus*	-	1870	2582299
Myxoma virus	*Oryctolagus cuniculus*	*Oryctolagus*	70	86917
Rabbit fibroma virus	*Oryctolagus cuniculus*	*Oryctolagus*	70	86917
Ectromelia virus	*Mus musculus*	-	22626	33882315
Variola virus	*Homo sapiens*	-	22454	34617928
Molluscum contagiosum virus	*Homo sapiens*	-	22454	34617928
Canarypox virus	*Passeriformes*	-	43	56253
Melanoplus sanguinipes entomopoxvirus	*Melanoplus sanguipines*	*Melanoplus*	0	0
Fowlpox virus	*Fowl*	*Gallus gallus*	17526	30024643
Amsacta moorei entomopoxvirus 'L'	*Amsacta moorei*	*Lepidoptera*	899	1135214
Lumpy skin disease virus	*Bos taurus*	-	1870	2582299
Mule deer poxvirus	*Odocoileus hemionus*	*Odocoileinae*	7	6369
Vaccinia virus	*Homo sapiens*	-	22454	34617928
Heliothis zea virus 1	*Heliothis zea*	*Lepidoptera*	899	1135214
